# Pan‐Genomic and Phenotypic Characterisation of Petroleum Hydrocarbon Degradation by *Pseudomonas* Species

**DOI:** 10.1111/1758-2229.70300

**Published:** 2026-02-15

**Authors:** Xiaopeng Guo, Shuhua Zhu, Ning Zhu, Shuhan Zhang, Shenghui Yang, Guanghong Luo, Hongbin Li, Yonggang Wang, Jing Sun, Borong Ma

**Affiliations:** ^1^ School of Life Science and Engineering Lanzhou University of Technology Lanzhou China; ^2^ Gansu Kaiyuan Biotechnology Development Center Co., Ltd Zhangye China; ^3^ Gansu Lanfei Environmental Protection Technology Co., Ltd Lanzhou China

**Keywords:** comparative genomics, complementarity of degrading enzyme systems, individuality and commonality, petroleum hydrocarbon degradation, *Pseudomonas* strains

## Abstract

*Pseudomonas*, a cornerstone genus in petroleum hydrocarbon bioremediation, exhibits remarkable metabolic diversity. To systematically decipher the genetic basis of this trait, we constructed a curated collection of representative *Pseudomonas* strains with documented degradation capabilities through a bibliometrics‐driven approach. Comparative genomic analysis revealed that these strains possess a rich repertoire of genes linked to petroleum hydrocarbon degradation, including those encoding key enzymes such as monooxygenases, dioxygenases, alcohol dehydrogenases, cytochrome P450, ferredoxins, and regulatory proteins (e.g., LuxR, AraC, GntR). Among the strains examined, *P. citronellolis* and *P. putida* contained the highest abundance of such genes. The accessory genome size varied considerably across the 15 strains (ranging from 3290 to 5745 genes), and functional enrichment analysis indicated a significant concentration of degradation‐related genes within this component. This genomic architecture not only reflects distinct metabolic specialisations among species but also implies potential synergistic interactions, as suggested by the broader genetic accessibility to polycyclic aromatic hydrocarbon (PAH) degradation pathways observed in *P. aeruginosa, P. luteola*, and *P. putida* . Overall, this study establishes a robust genomic framework that extends beyond single‐species analysis, offering a genus‐level perspective essential for designing tailored, high‐efficiency microbial consortia for targeted bioremediation strategies.

## Graphical abstract

*Pseudomonas* is one of the most significant bacterial genus involved in the petroleum hydrocarbon bioremediation. Innovatively, through pan‐genomic analysis at the genus level, we found that different *Pseudomonas* strains have excellent complementarity at the nodes of the degradation pathway of petroleum hydrocarbons.

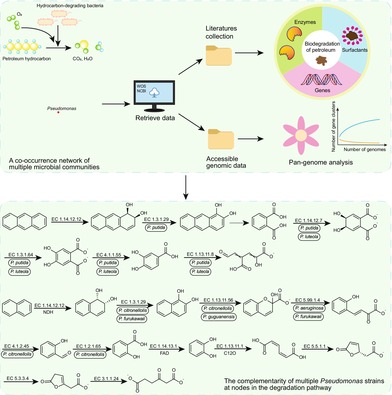

## 1 Introduction

The utilisation of petroleum energy and related industries has facilitated socioeconomic development, yet it has also led to significant environmental pollution. Microbial remediation stands as one of the most effective and environmentally sustainable technologies for restoring petroleum‐contaminated environments. *Pseudomonas*, a well‐studied bacterial genus, demonstrates a strong capacity for petroleum hydrocarbons degradation, with numerous degradation‐related genes having been identified across its species. For instance, the genome‐assembled strain *P. aeruginosa* DN1 carries more than 100 candidate genes associated with polycyclic aromatic hydrocarbon (PAH) degradation (He et al. 2018). Moreover, *P. fluorescens* AH‐40 was reported to degrade 97% of phenanthrene (150 mg·L^−1^) within 15 days, a capacity linked to increased copy numbers of the degradation genes *nahAc* and *c23o* (Mawad et al. 2020). Notably, disruption of key genes (e.g., *catA, pcaG, pcaH, rhdA*) did not completely abolish fluorescent anthracene degradation, indicating that considerable potential remains for discovering new degradative genes in *Pseudomonas*.

Despite the extensive repertoire of degradative gene in *Pseudomonas*, the links between genotype and phenotype are often inadequately characterised. Current research also tends to focus on single species, overlooking functional integration at the genus level, which limits the stability of interspecies synergies. In reality, microbial communities constitute complex microecosystems shaped by nutrient symbiosis, metabolic cross‐feeding, quorum sensing, and horizontal gene transfer. Therefore, a systematic analysis of the degradation traits and genetic foundations across diverse strains can offer deeper insights into the functioning of microecosystem. Such an approach would help maximise the individual degradation potential of strains and guide the design of efficient and stable synthetic consortia. For example, *P. fluorescens* utilises both short‐ and long‐chain n‐alkanes (Xiang et al. 2023), *P. putida* degrades benzene, toluene, o‐xylene, and PAHs (Kneubehl and Iyer 2023; Crosby and Stadler 2025), and *P. aeruginosa* efficiently metabolises n‐hexadecane and paraffins in crude oil (Stancu 2023). This functional diversity underscores the important role of *Pseudomonas* in high‐performance petroleum‐degrading microbial communities. Two strategies are used to construct petroleum hydrocarbon‐degrading microbial communities. The conventional top‐down approach adapts natural communities through ecological selection to achieve rapid functional gains. However, it offers limited insight into internal interactions and metabolic networks, hindering fine‐tuning across contexts. In contrast, the bottom‐up approach addresses these limitations. It first assembles a library of petroleum‐degrading strains, then applies microscale regulation to resolve metabolic patterns and interactions. This process culminates in interaction‐driven metabolic models that enable rational design and molecular‐level control of community function and stability. For instance, Goma‐Tchimbakala et al. used metagenomics to design a metabolically complementary synthetic community that produced surfactants and degraded crude oil, achieving up to 40.6% degradation of aromatic compounds (Goma‐Tchimbakala et al. 2022). Ruan et al. developed superCC, a metabolic modelling framework for assembling communities based on interaction patterns inferred from natural microbiomes (Ruan et al. 2024). Similarly, a genome‐scale metabolic model (GSMM) of *P. fluorescens* has guided metabolic engineering strategies to enhance catechol degradation (Huang and Lin 2020). Collectively, these examples show that effective bottom‐up construction relies on deep genetic characterisation of individual strains, facilitating the development of models that objectively assess strain individuality and complementarity.

This study aims to systematically elucidate functional complementarity among *Pseudomonas* species in petroleum hydrocarbon degradation by integrating genomic and phenotypic evidence. We began with a bibliometric survey of the Web of Science core collection to identify representative *Pseudomonas* strains with available genomes. Innovatively, we then extended the pan‐genome concept—typically confined to single species—to multiple species within the genus. Using a comparative genomics framework, we evaluated strain‐specific, shared, and complementary capacities in degrading petroleum hydrocarbons. Based on these results, we propose a genus‐centric strategy for designing synthetic microbial consortia. The novelty of this research is threefold: (1) extending pan‐genome analysis across a genus to leverage native co‐culture stability and identify shared and unique genomic features within a “common‐yet‐distinct” framework; (2) integrating genotype–phenotype evidence to reveal complementary oil‐degrading traits among *Pseudomonas* species and high‐quality genetic determinants of degradation; and (3) establishing a genus‐centric design principle for building highly synergistic consortia. As synthetic microbiology advances, optimising community structure at the genus level is expected to enhance the stability of core functional taxa and improve the efficacy of multi‐strain microbial agents.

## 2 Acquisition and Processing of the Literature Set and Genome Data

### 2.1 Establishment of the Literature Set, Statistics of Degrading Strains, and Acquisition of Genome Data

**FIGURE 1:**
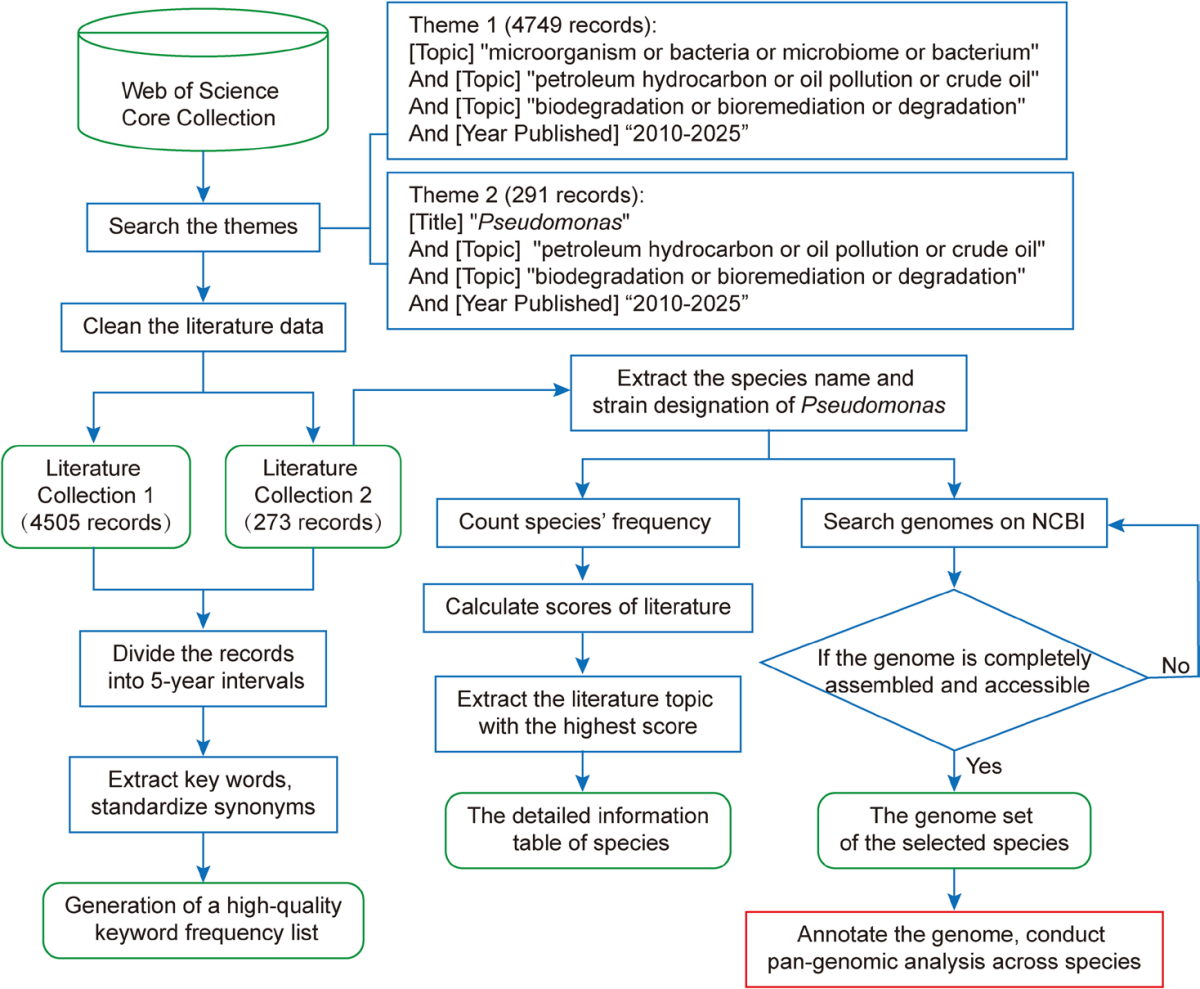
Schematic of the literature retrieval and analysis strategy. The workflow illustrates the construction of a local literature dataset, frequency analysis of keywords, and the application of specific criteria for selecting *Pseudomonas* strains and genomes.

Relevant literature on the biodegradation of petroleum hydrocarbons by microorganisms was retrieved from the Web of Science (WOS) Core Collection. The primary search query was: “[Topic] microorganism or bacteria or microbiome or bacterium” and “[Topic] petroleum hydrocarbon or oil pollution or crude oil” and “[Topic] biodegradation or bioremediation or degradation”. To specifically gather literature on petroleum hydrocarbon degradation by the genus *Pseudomonas*, an additional search was conducted using the terms: “[Title] *Pseudomonas*” and “[Topic] petroleum hydrocarbon or oil pollution or crude oil” and “[Topic] biodegradation or bioremediation or degradation” (Figure 1). The search encompassed publications published between January 1, 2010, and December 31, 2025.

Information pertaining to literature citation frequency, strain research prominence, and strain degradation characteristics was analysed using HistCite Pro 2.1, CiteSpace II 6.3.R1 (64‐bit), and Microsoft Excel 2019. To help assess the significance of individual strains, a citation score was calculated for each key article containing a degrading strain using the formula: score = (the citation frequency)/(the number of records during publication year−current year). This score is also used to help assess the significance of a strain.

Strain selection followed these criteria: (1) strong petroleum hydrocarbon degradation performance reported in Web of Science (WOS) literature; (2) high citation frequency within local literature collections; (3) availability of high‐quality, complete, openly accessible genomes on NCBI; and (4) inclusion of diverse species to ensure representativeness. All genomic data were obtained exclusively from the NCBI database and met essential requirements, including explicit author declaration permitting data citation and reuse, public accessibility, and complete high‐quality assemblies. For a limited number of strains noted for relevant degradation traits but lacking de‐novo whole‐genome assemblies, we substituted conspecific genomes with the highest assembly quality, the largest genome size, and openly shared data. In total, 15 strains that satisfied these criteria and exhibited outstanding petroleum hydrocarbon degradation capabilities were included in the study. These strains belong to 11 different species, including *P. aeruginosa, Pseudomonas citronellolis, P. fluorescens, Pseudomonas furukawaii, Pseudomonas guguanensis, Pseudomonas luteola, Pseudomonas monteilii, Pseudomonas nitroreducens, P. putida, Pseudomonas stutzeri, Pseudomonas veronii*.

### 2.2 Genome‐Wide Annotation of Genes Associated With Petroleum Hydrocarbon Degradation

Genome‐wide annotation of genes associated with petroleum hydrocarbon degradation was performed. A phylogenetic tree was constructed using the Neighbour‐Joining (NJ) method in MEGA software to compare the evolutionary relationships among 15 bacterial strains. Gene sequence annotation was carried out with Prokka software and the ggplot2 package in R studio. Degradation‐related genes were compiled based on literature reports as well as functional annotations from multiple databases, including GO, COG, KEGG (assigned via orthology mapping), NR, SwissProt, TrEMBL (assigned via sequence similarity), and Pfam (assigned via domain structure). The abundance, density, and proportion of degradation genes in each strain were visualised using TBtools software (Chen et al. 2023).

### 2.3 Comparative Genomic Analysis

In addition to the intuitive comparison of the number and functional categories of genome‐wide degradation genes, this study innovatively applies the concept of “pan‐genome” to conduct a comprehensive analysis and comparison among the genomes of hydrocarbon‐degrading bacteria within the *Pseudomonas* genus. Using multiple comparative tools (Roary, PanOCT, Panaroo, PanX, OrthoFinder, OrthoMCL), we generated a gene presence‐absence matrix. By performing direct homology analysis of sequences in GFF files using BLASTp, we identified both variable and core components of the genome. Genes that are present in 11 or more strains are classified as part of homologous gene clusters (68.75%). Finally, the R packages “devtools” and “RIdeogram” were utilised for collinearity analysis and visualisation, revealing conserved sequences and rearrangement events between the genomes of different strains.

### 2.4 Bibliometric‐Based Co‐Occurrence Network Construction of Microbial Communities Containing *Pseudomonas*

To construct the co‐occurrence network, we first identified the 100 most cited studies from a targeted search in the Web of Science that reported on petroleum‐degrading microbial communities containing *Pseudomonas*. After extracting the abstracts and annotating the microbial taxa, we generated a co‐occurrence matrix using R (v4.3.1; igraph), based on joint reporting frequency. This matrix was then visualised in Gephi (v0.10) (Bastian et al. 2009) using the ForceAtlas2 layout (scaling factor 10.0, overlap prevention), which unveiled key taxonomic associations within these communities.

### 2.5 Investigation of Potential Complementarity Among Different Strains by Bibliometrics, Comparative Genomes and Community Co‐Occurrence

Firstly, bibliometric analysis was employed to preliminarily assess differences in petroleum hydrocarbon degradation traits among the selected *Pseudomonas* strains. Subsequently, genome‐wide comparative analysis based on functional annotations further elucidated strain‐specific biodegradation capabilities. By adopting a pan‐genome approach, the distribution of degradation‐associated genes in the core and accessory genomes was also revealed. Finally, potential metabolic cooperation between strains was explored through pathway enrichment analysis. Together, these approaches uncovered complementary genetic foundations for petroleum hydrocarbon degradation across different strains, integrating phenotypic, genomic, and metabolic perspectives.

## 3 Discussion on the Phenotypic Characteristics, Genetic Bases, and Potential Complementarity Among Petroleum Hydrocarbons‐Degrading Strains From Multiple *Pseudomonas* Species

### 3.1 Evolution of Keywords, Reported Frequency of Strains, and Degradation Characteristics as Reflected in the Literature

#### 3.1.1 Significance of *Pseudomonas* Strains on Petroleum Hydrocarbon Biodegradation as Indicated by Keyword Evolution

Keyword evolution analysis with five‐year time slices and frequency statistics on two literature datasets showed consistent research hotspots. One set covers general microbial degradation of petroleum hydrocarbons, and the other focuses specifically on *Pseudomonas* strains. High‐frequency keywords like “community/consortium”, “genes/expression”, and “surfactant/biosurfactant” appear in both research topics. It highlights a shared focus on microbial cooperation, especially for degrading recalcitrant PAHs, a process in which *Pseudomonas* species are key. Biosurfactant production has also been identified as an important indicator for evaluating the degradation capability of microbial strains or communities.

**FIGURE 2:**
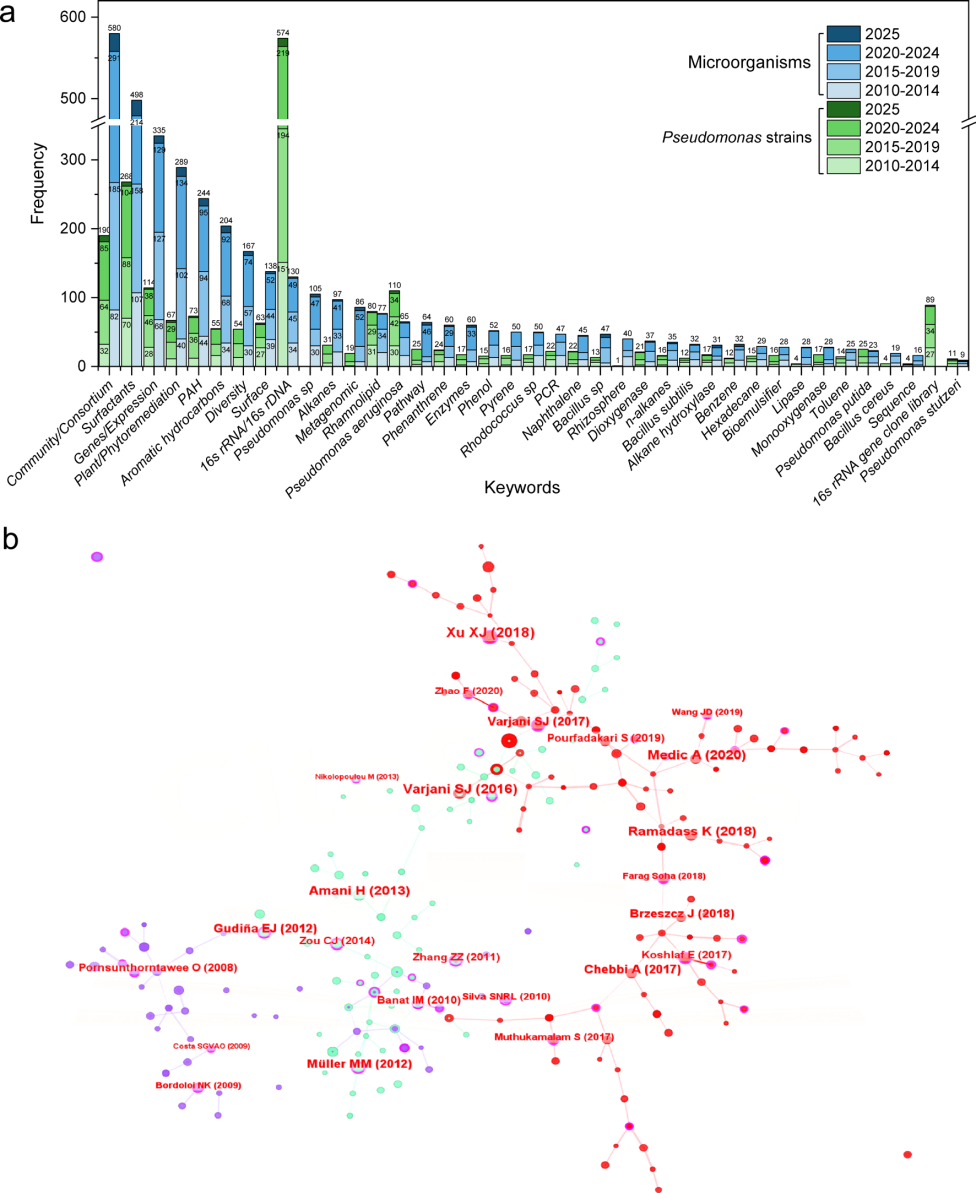
(a) Keyword evolution in the literature pertaining to petroleum hydrocarbon biodegradation by various microorganisms (blue bars) and specifically by *Pseudomonas* strains (green bars). (b) Literature co‐citation network in the field of biodegradation of petroleum hydrocarbons by *Pseudomonas*.

Among the genus‐level keywords in the general petroleum hydrocarbon degradation theme, *Pseudomonas* sp., *P. aeruginosa, Rhodococcus* sp., and *Bacillus* sp. rank highest in frequency (Figure 2a). The total frequency of keywords containing “*Pseudomonas*” exceeds 200, significantly surpassing that of other genera, highlighting the considerable degradation potential of various *Pseudomonas* species. *P. aeruginosa* is the most frequently mentioned species in both datasets. Other notable species include *P. putida* , which is particularly effective in degrading C14–C32 alkanes (Zheng et al. 2018), as well as *P. stutzeri* and *P. flavus*, which exhibit high growth rates on crude oil hydrocarbons (El‐Sheshtawy et al. 2014). The high frequency of *B. subtilis* keywords in the general literature set suggests potential synergistic effects with *Pseudomonas*, supported by studies where co‐culture of *B. subtilis* and *P. aeruginosa* enhanced crude oil degradation (Wu et al. 2023).

At the molecular level, “16S rRNA/16S rDNA” ranks highly in both datasets, reflecting the continued importance of 16S‐based sequencing in microbial diversity studies. Keywords like “genes/expression,” “genes,” and “pathway” are prominent in both 2015–2019 and 2020–2024 periods, highlighting ongoing interest in genetic mechanisms and metabolic pathways involved in degradation (Figure 2b). These insights help identify efficient strains and guide the design of synthetic microbial communities for bioremediation.

#### 3.1.2 Reported *Pseudomonas* Species With Petroleum Hydrocarbon Degradation Ability and Their Degradation Characteristics

Beyond the keyword analysis, the local literature set reveals a distinct ranking of *Pseudomonas* species based on their citation frequency. *P. aeruginosa* and *P. putida* demonstrate the highest mention, underscoring their documented prominence in the biodegradation of petroleum hydrocarbon pollutants. Previous studies highlight that *P. aeruginosa* exhibits robust metabolic versatility and broad environmental adaptability, while *P. putida* shows proven efficacy in degrading compounds such as phenol and C14–C32 alkanes (Emmarloo et al. 2024; Zheng et al. 2018). As shown in Table 1, multiple *Pseudomonas* species beyond those previously mentioned have been confirmed to degrade petroleum hydrocarbons, including *P. stutzeri, P. fluorescens, P. citronellolis, P. pseudoalcaligenes, P. nitroreducens, P. veronii, P. antarctica, P. cepacian, P. guguanensis, P. otitidis, P. furukawaii, P. luteola*, and *P. monteilii* . Their degradation potential is continually being explored. For instance, *P. citronellolis* strain SJTE‐3 efficiently degrades long‐chain alkanes and aromatic compounds, achieving removal rates of 86%, 85%, and 88% for *n*‐dodecane, *n*‐tetradecane, and naphthalene, respectively (Koutinas et al. 2021). Table 1 summarises the degradation traits of each strain based on highly cited representative studies, underscoring the functional diversity within the genus. Although less frequently reported, species such as *P. furukawaii* and *P. nitroreducens* exhibit notable capabilities in degrading specific pollutants, particularly benzene‐series compounds (Mawad et al. 2020; Mohapatra and Phale 2021). For example, the degradation rates of *P. furukawaii* PPS‐19 for *n*‐dodecane and pyrene can reach as high as 94.5% and 83%, respectively (Vandana and Das 2023). Furthermore, *P. nitroreducens* not only produces surfactants but also shows tolerance to saturated hydrocarbons such as *n*‐heptane, *n*‐decane, *n*‐pentadecane, and *n*‐cetane (Stancu 2023). These findings illustrate that different *Pseudomonas* species possess distinct degradation profiles. When constructing synthetic microbial communities, targeted combinations of strains can broaden or fine‐tune the degradation spectrum, leveraging their complementary strengths to enhance bioremediation efficiency.

**TABLE 1:** Occurrence frequency and degradation characteristics of *Pseudomonas* strains that degrade petroleum hydrocarbons in the literature.

*Pseudomonas* Strains	Freq ranking	Literature examples with high scores			Characterisation of petroleum hydrocarbon degradation by different *Pseudomonas* species			
		Theme elaboration	LCS	GCS	Degradation substrate	Degradation efficiency	Biosurfactant Production	Other
*P. aeruginosa*	1	Extracellular enzymes and surfactants play crucial roles in the biodegradation of crude oil (Muthukumar et al. 2022).	35	35	PAH, medium‐ and long‐chain *n*‐alkanes (C12‐C32), salt tolerance (Muthukumar et al. 2022).	48.23%; co‐culture 63.05% (Nnabuife et al. 2022; Wu et al. 2023)	Confirmed (Medic et al. 2020).	
*P. stutzeri*	2	The effect of the surfactant alkyl polyglycoside (APG) on the enhanced biodegradation of petroleum hydrocarbon‐contaminated soil (Li, Huang, et al. 2020).	45	47	A wide range of substrates (Fan et al. 2024).			Strong adaptability to environmental conditions, diversity in metabolic pathways, and significant salt tolerance (Fan et al. 2024).
*P. putida*	2	The immobilisation of *P. putida* P53 in wood chips embedded in sodium alginate beads was utilised to degrade high concentrations of phenol (Abarian et al. 2019).	33	34	Phenol, hexadecane, anthracene, and naphthalene (Abarian et al. 2019)	37.83% (Li et al. 2025); mixed consortia 86% (Parthipan et al. 2017)	Confirmed (Sajna et al. 2015).	
*P. fluorescens*	3	The effect of the addition of exogenous surfactants on hydrocarbon biodegradation and the properties of cell surfaces (Kaczorek and Olszanowski 2011).	47	52	Phenanthrene, naphthalene (Kaczorek and Olszanowski 2011).		Rhamnolipids (Mawad et al. 2020).	Thermal resistance, biocontrol.
*P. citronellolis*	4	*P. citronellolis* SJTE‐3 enhances the biodegradation and acidification of drilling wastewater through the simultaneous production of biosurfactants and polyhydroxyalkanoates (Koutinas et al. 2021).	6	8	Aromatic hydrocarbons, including fluorenes (Oyehan and Al‐Thukair 2017).	83% (Baig et al. 2022)	Confirmed (Koutinas et al. 2021).	
*P. pseudoalcaligenes*	5	The impact of external mass transfer on the degradation of fluorene (a polycyclic aromatic hydrocarbon) by immobilised low‐density polyethylene (LDPE) in a packed bed bioreactor (PBBR) was analysed (Sonwani et al. 2019).	17	17	PAH (Sonwani et al. 2019).		Lipopeptide bioemulsifiers (Mawad et al. 2021).	The petroleum degradation rate exceeds 90% (Sonwani et al. 2019).
*P. nitroreducens*	5	The potential of bioemulsifiers for bioremediation and enhanced oil recovery of hydrocarbons in marine environments has been demonstrated (de Sousa and Bhosle 2012).	45	48	Naphthalene (NP) and other natural aromatic compounds; Polyethylene terephthalate (PET) (Mohapatra and Phale 2021).	22.9%–70% (Totubaeva et al. 2019)		
*P. veronii*	5	*P. veronii* 7–41 is capable of growing on medium‐chain n‐alkanes (C8‐C12) and polycyclic aromatic hydrocarbons, such as naphthalene (Mullaeva et al. 2022).	5	5	Diesel oil (Mullaeva et al. 2022).			The synthesis of fatty acid esters (Khoury et al. 2011).
*P. antarctica*	5	Production and characterisation of lipopeptide biosurfactants from a novel strain of *P. antarctica* 28E utilising crude glycerol as a carbon source (Ciurko et al. 2023).	3	3	Terminal *n*‐alkane oxidation activity (Wang et al. 2021).		Confirmed (Ciurko et al. 2023).	Cold‐resistant
*P. cepacia*	5	Characterisation of the biosurfactant produced by *Pseudomonas* CCT6659 in the presence of industrial waste and its application in the biodegradation of hydrophobic compounds in soil (Silva et al. 2014).	80	82		52.5%; consortium 97.5% (Mawad et al. 2021)	Glycolipid biosurfactants (Silva et al. 2017).	
*P. guguanensis*	6	Optimisation of bioprocess parameters to enhance the yield of monorhamnolipid by *P. guguanensis* marine (RamyaDevi et al. 2018).	7	9	Hexadecane, diesel, and crude oil (Vajiravelu et al. 2021).		Monorhamnolipid (1264.52 Da) (RamyaDevi et al. 2018).	
*P. otitidis*	6	Bioremediation of engine oil‐contaminated soil and water using the *P. otitidis* strain DU13, along with the characterisation of its biosurfactant (Gogoi et al. 2022).	2	2		20.0%–82.4% (Yan et al. 2023)	Confirmed (Gogoi et al. 2022).	Enhance seed germination and promote plant growth (Cuong et al. 2023).
*P. furukawaii*	6	Cell surface hydrophobicity and the degradation of petroleum hydrocarbons by the biofilm‐forming marine bacterium *P. furukawaii* PPS‐19 under various physicochemical stresses (Vandana and Das 2023).	5	5	Pyrene, *n*‐dodecane and following a single‐terminal oxidation pathway (Vandana and Das 2023).			High cell surface hydrophobicity
*P. luteola*	6	Effects of a novel oil‐degrading bacterium, *P. luteola* PRO23, on diesel fuel degradation and biosurfactant production (Atanaskovic et al. 2016).	2	4			Confirmed (Agwu et al. 2013).	
*P. monteilii*	6	The combined use of *P. nitroreductans* S8 and *P. montelli* S17 demonstrated a synergistic effect in the degradation of the PET surface through their colonisation behaviour, thereby enhancing the subsequent degradation of PET (Yan et al. 2023).	2	2	PAH; Polyethylene terephthalate (PET) (Alessandrello et al. 2017).	18.8% (Shukor et al. 2009)		Stress resistance and tolerance to both salt and alkaline (Alessandrello et al. 2017).

*Note:* Data sourced from the Web of Science core collection.

Abbreviations: GCS, Global Citation Score; LCS, Local Citation Score.

### 3.2 Key Molecules and Regulatory Mechanisms in Degradation of Petroleum Hydrocarbons by *Pseudomonas* Strains

#### 3.2.1 Characterisation of Surfactants Secreted by *Pseudomonas* and Their Biosynthetic Mechanisms

**FIGURE 3:**
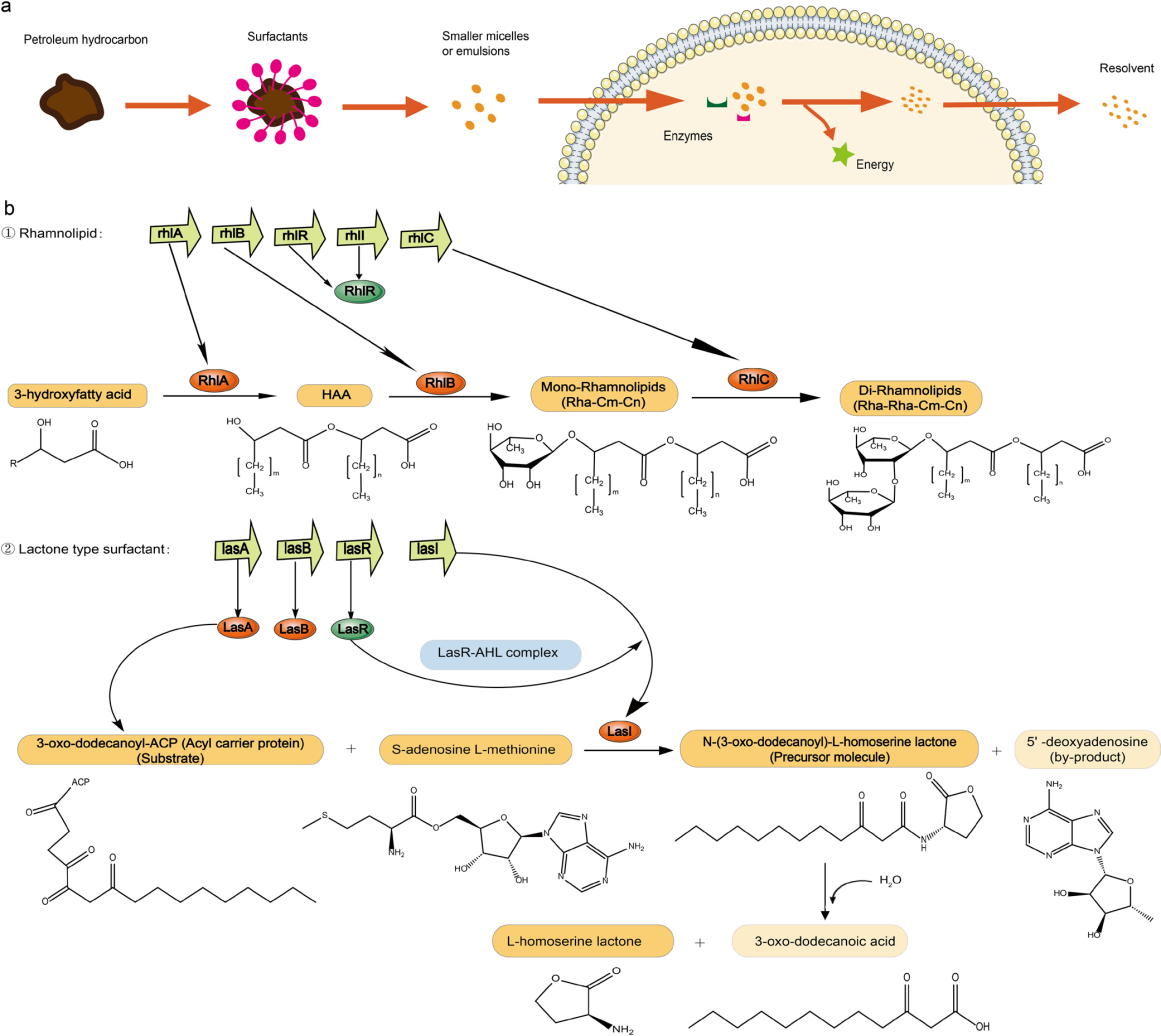
Major surfactants secreted by *Pseudomonas* and their biosynthetic mechanisms. (a) Mechanism by which biosurfactants facilitate the microbial degradation of petroleum hydrocarbons. (b) Clusters of genes encoding enzymes involved in the synthesis of two typical surfactants, along with the chemical reaction processes catalysed by these enzymes. In the schematic, orange boxes represent enzymes, while light yellow boxes denote chemical intermediates.

As revealed by keyword analysis, biosurfactants play a crucial role in the microbial degradation (Figure 3a) of petroleum hydrocarbons (Li, Huang, et al. 2020; Muthukumar et al. 2022). Among them, Rhamnolipids and lactonic sophorolipids exhibit synergistic effects due to their complementary structures and functions. Rhamnolipids effectively emulsify hydrocarbons into bioaccessible droplets (Phulpoto et al. 2022; Li, Wang, Zhou, et al. 2024), while lactonic compounds modulate microbial community behaviours via quorum sensing interference (Elshikh et al. 2017). Their combined use creates a “physical solubilisation–signal regulation–metabolic activation” cascade, significantly enhancing petroleum hydrocarbon degradation (Song et al. 2013).

The synthesis of these surfactants is genetically regulated in *Pseudomonas*. Key gene clusters such as *rhl* system control rhamnolipid production (Figure 3b), where *rhlA* and *rhlB* are co‐transcribed and the RhlA enzyme determines structural specificity through dual sulfatase‐acyltransferase activity (Tang et al. 2023; Kunzler et al. 2024). Genes including *rhlAB* and *rhlC* have been employed to construct engineered *P. aeruginosa* strains with high biosurfactant yields (Wang et al. 2007). Simultaneously, *lasAB* and *lasR* genes govern lactone surfactant synthesis (Li et al. 2023). Furthermore, rhamnolipids cooperate with lipopeptides from *Bacillus* to form mixed micelles (Zhou et al. 2023), and can engage in cross‐talk with AHL lactones to co‐regulate biofilm formation and degradation gene expression (Du et al. 2015).

This mechanistic synergy is leveraged in practical bioremediation. High‐yielding *Pseudomonas* strains serve as bioaugmentation agents, while fermented rhamnolipid products act as biostimulants, collectively accelerating pollutant removal in soil and water systems (Nie et al. 2010; Tayeb et al. 2022; Zhou et al. 2022). The integration of genetic regulation, multi‐surfactant collaboration, and application strategies provides an efficient solution for petroleum hydrocarbon contamination remediation.

#### 3.2.2 Strongly Correlated Enzyme Systems Involved in Petroleum Hydrocarbon Degradation

*Pseudomonas* strains exhibit considerable degradation of components such as long chain alkanes and aromatic hydrocarbons, primarily due to their genetic repertoire to encode various degradation‐related enzymes (Table 2). The process typically initiates under chemoaerobic conditions. For long‐chain *n*‐alkanes, two classical aerobic metabolic pathways are recognised. (1) Long‐chain alkanes are catalysed by cytochrome P450 or long‐chain alkane hydroxylase (LadA) to become primary alcohols, and then catalysed by alcohol dehydrogenase and aldehyde dehydrogenase to generate carboxylic acids (Okoye et al. 2024). It's followed by activation to acyl‐CoA for β‐oxidation (Dong et al. 2024). (2) Subterminal alkane monooxygenase catalyses the formation of secondary alcohols (Libisch 2024), which are dehydrogenated to ketones and then converted to esters via Baeyer‐Villiger monooxygenase; ester hydrolysis yields intermediates that enter central carbon metabolism (Gregson et al. 2018; Kirschner et al. 2007). Under anaerobic conditions, alkane activation shifts to carboxylation at the C‐3 position using nitrate or sulphate as electron acceptors, with subsequent rearrangement and β‐oxidation (Dong et al. 2024). Critically, oxidases encoded by genes such as those for cytochrome P450 oxidase (degrading C5–C10 alkanes) (Costa and Millán 2024), alkane hydroxylase AlkB (C5–C16 alkanes) (Libisch 2024), flavin‐binding monooxygenase AlmA (C10–C30 alkanes), long‐chain alkane hydroxylase LadA (C15–C36 alkanes) (Okoye et al. 2024) play pivotal roles, often with genomic multiplicity enhancing degradation efficiency.

**TABLE 2:** Substrates and catalytic properties of essential enzymes in petroleum hydrocarbon degradation in *Pseudomonas*.

Degrading enzymes	Enzyme abbreviation	Degradation substrate	Catalytic characteristic	References
Particulate methane monooxygenase	pMMO	C1–C4	Maintain high electron transfer efficiency at low temperatures	(Lu et al. 2025)
Soluble methane monooxygenase	sMMO	C1–C9	Radical rebound mechanism and coordinated oxygen insertion mechanism	(Sang et al. 2025)
Butane monooxygenase	BMO	C2–C9	High efficiency, high selectivity, synergistic with other enzymes	(Jin et al. 2025)
Binding membrane non‐heme diferric alkane hydroxylase	AlkB	C5–C12	Extensive substrate adaptability, high efficiency, and structural features integrated with membranes	(Schlechter et al. 2025)
Cytochrome P450 monooxygenase	CYP153	C5–C16 and long chain alkanes	High stereoselectivity and regional selectivity, high selectivity under mild conditions	(Eltoukhy et al. 2022)
Flavin‐dependent monooxygenase	AlmA	C18–C38	Catalyse the reaction of ketone to ester; Catalyse the oxidation of sulphur and other heteroatoms	(Zhao et al. 2025)
Long chain alkane degrading enzyme	LadA	C15–C36	Terminal oxidation	(Sandholm et al. 2025)
Non‐heme iron integrated membrane alkane hydroxylase	AlkM	C12–C44	Key enzyme for initiation of alkane degradation	(Sun et al. 2014)
Alkyl succinate synthase/Methylalkyl succinate synthase	ASS/MAS	C3–C50	A key enzyme in anaerobic toluene degradation, promoting fumaric acid addition reaction	(Ji et al. 2019)
Naphthalene carboxylase	NaphC	Naphthalene	Through the synergistic action with other enzymes such as 2‐naphthalene coA reductase, the reduction and degradation of naphthalene ring are further promoted, so as to achieve effective degradation of naphthalene and other polycyclic aromatic hydrocarbons	(Madison et al. 2022)
Benzyl succinate synthetase	BSS	Toluene	During the initial degradation of toluene, fumaric acid is converted to benzyl succinate	(Cabral et al. 2022)
Catalase	KatB	Hydrogen peroxide	Remove and neutralise hydrogen peroxide to reduce oxidative stress	(Yang et al. 2023)
Lipase	ClpS	Aliphatic alcohol	Beta ‐ oxidation, degradation of intermediate metabolites	(Duan et al. 2025)
Oxidoreductase	OR	Alkanes, cycloalkanes and aromatic hydrocarbons	Degrade intermediate metabolites	(Bui et al. 2025)
Naphthalene dioxygenase	NDO	Naphthalene	High specificity and high degradation rate	(Davoodi et al. 2023)
Hydroxylase	NahG, NahU	Long chain alkanes	A hydroxyl group is added to the end of the alkane molecule	(Costa et al. 2019)
Alcohol dehydrogenase	ADH	Alcohol	Proton transfer and hydride transfer	(Gogoi et al. 2025)
Oxidase	LadA	Naphthene, aromatic hydrocarbon	Ring‐opening reaction	(Chlebek et al. 2022)
Dehydrogenase	DH	Hydroxy hydrocarbons	Proton transfer and hydride transfer	(Moni et al. 2025)

The degradation of aromatic hydrocarbons is hindered by the resonance‐stabilised aromatic ring, yet *Pseudomonas* species efficiently catabolise these compounds through specialised oxygenase‐ and dehydrogenase‐mediated pathways. Initial ring hydroxylation typically yields cis‐dihydrodiols, which are rearomatized to catechols; subsequent ring cleavage produces dicarboxylic acid intermediates (Zavala‐Meneses et al. 2024). Key enzymes include aryl alcohol dehydrogenases, salicylate hydroxylase, naphthalene dioxygenase (NDO), and catechol dioxygenases (Li, Liu, Zhang, et al. 2024; Pandolfo et al. 2024). For example, phenol hydroxylase and indole hydroxylase in *Pseudomonas* are involved in phenol degradation (Nagayama et al. 2015), while benzylsuccinate synthase catalyses the initial degradation of toluene. Catechol, a central intermediate, undergoes ring‐opening via catechol 1,2‐dioxygenase or catechol 2,3‐dioxygenase; the latter can be rate‐limiting due to oxidative inactivation of its Fe^2+^ cofactor Fe^2+^ oxidation (Zhou et al. 2019; Jiang et al. 2024; Celesia et al. 2017). Laccase is a copper‐containing polyphenol oxidase that relies on the synergistic electron transfer of copper ions in its active center to catalyse the oxidation of phenolic and non‐phenolic substrates. In contrast, NDO incorporates dioxygen directly into aromatic rings to form cis‐dihydrodiols (Choukairi Afailal et al. 2024). The genetic diversity underlying these enzyme systems, including multiple homologues and pathway variants, enables functional complementarity and adaptive degradation across *Pseudomonas* species, highlighting a direct link between genomic plasticity and phenotypic versatility in aromatic hydrocarbon metabolism.

#### 3.2.3 Transcriptional Regulatory Mechanisms Associated With Petroleum Hydrocarbon Degradation by *Pseudomonas* Strains

The expression and secretion of degrading enzymes in *Pseudomonas* strains involve complex transcriptional regulation mechanisms, often resulting from the integrated action of multiple signalling pathways in response to specific environmental factors (Figure S1). Key transcription factors like *alkS*, *phnR*, *pahR*, and *nahR* specifically regulate clusters for alkane, BTEX, PAH, and naphthalene degradation, respectively (Li, Liu, Liu, et al. 2024). For instance, AlkS links to styrene oxidase activity, while *nahR* activates naphthalene pathways via salicylate induction (Malhotra et al. 2023), a mechanism that has been exploited in biosensor design. Furthermore, the global transcriptional regulators including LuxR/MalT, AraC/XylS, and GntR families enable broad adaptability: LuxR senses substrate signals, AraC/XylS combines DNA‐binding with substrate recognition, and GntR responds flexibly to metabolites, also influencing cell motility and antibiotic resistance. It is worth noting that redox reactions are central to biodegradation, as haemoglobin‐catalysed H_2_O_2_ oxidation boosts TPH degradation from 26% to 76% (Jho et al. 2016). *Pseudomonas* modulates oxidoreductase expression via redox‐sensing proteins (Gong et al. 2015), inducing nitrate/nitrite reductases under hypoxia and catalase/superoxide dismutase in response to oxidative stress (Zhang et al. 2021).

In microecology settings, shifts in pollutants composition and concentration, substrate structure, oxygen availability, nutrient status, and temperature can signal unfavourable conditions to *Pseudomonas*. In response, these bacteria activate specific transcriptional regulatory mechanisms to adapt. First of all, petroleum hydrocarbons are typically non‐preferred, recalcitrant carbon sources, and such environments often coincide with nitrogen and phosphorus limitation. To optimise the uptake and utilisation of scarce nutrients, *Pseudomonas* modulates genes for nutrient acquisition and assimilation (e.g., glutamine synthase, phosphate transporters) (Pacwa‐Plociniczak et al. 2024). This regulation sustains catabolism under adverse conditions, and the synthesis or activity of petroleum hydrocarbon degrading enzymes can be promoted. For example, *P. putida* positively regulates the transcription of nitrogen assimilation‐related genes such as *glnA* and *rhlA*, thereby enhancing nitrogen supply to alleviate the inhibition of naphthalene 1, 2‐dioxygenase activity by low nitrogen conditions (Pozdnyakova‐Filatova et al. 2020). Compared with microbial remediation alone, one of the reasons why plant‐microbial combined remediation system has better degradation effect is that bacteria activate carbon metabolism in response to rhizosphere microecological factors such as root exudates. For *Pseudomonas*, organic acids, amino acids and sugars in root exudates act as signal molecules to regulate the expression of genes related to hydrocarbon degradation, significantly enhancing the degradation of TPHs (Chetverikov et al. 2021; Khazaei et al. 2021). The excellent redox ability of *Pseudomonas* strains is not only the core of degrading petroleum hydrocarbons, but also the key to coping with various stresses. For example, the catalase encoding genes *katA, katB, katE* and *katG* and the peroxide sensing gene *oxyR* are highly conserved in different *Pseudomonas* species, which can significantly improve the adaptability to stress such as salt tolerance and heavy metal resistance (Zhang et al. 2019). The role of new redox‐related genes in stress adaptation of *Pseudomonas* species is constantly being revealed. For example, the gene PA2580 in *P. aeruginosa* , encodes an NADPH‐quinone reductase, whose mutation increases sensitivity to hydrogen peroxide and reduces catalase expression (Zhou et al. 2019); production of diphenylamine peroxidase contributes to the survival of *P. putida* under adversity (Bucková et al. 2010). In addition, some *Pseudomonas* strains have great low temperature adaptability, which involves boosted ATP synthesis, cold‐shock proteins, and membrane fluidity adjustments (Ankenbauer et al. 2020; Wang et al. 2016; Yan et al. 2017), while salt stress triggers mannitol‐1‐phosphate dehydrogenase for osmotolerance (Habib et al. 2018).

Under high‐stress conditions, *Pseudomonas* strains alter global transcription related to adversity adaptation to enhance their viability (Elmassry Moamen et al. 2019). In addition to the previously mentioned families of transcription factors, such as LuxR/MalT, AraC/XylS, and GntR, *Pseudomonas*, which is classified as a Gram‐negative bacterium, contains the largest gene family encoding histidine kinases and response regulators that form a two‐component system. This system enables cells or populations to sense and respond to diverse environmental changes. For example, the expression of *gacS/gacA* is modulated by external signals such as external temperature, nitrogen source, salinity and oxygen concentration. Promoters of multiple genes involved in carbon/nitrogen metabolism, biofilm formation, motility, chemotaxis, transport, and stress responses carry conserved GacA‐binding motifs. When adverse environmental factors compromise cellular proliferation and metabolism, signalling molecules such as cAMP and (p)ppGpp regulate the expression of specific stress response genes (including heat shock protein gene *hsp*, cold shock protein gene *csp* and oxidative stress‐related gene *sod*) to maintain basal metabolic activity and facilitate entry into the viable but non‐culturable (VBNC) state, thereby promoting survival under harsh conditions. In practical applications, resuscitation of VBNC *Pseudomonas* can be achieved using physical, chemical, or biological methods. These methods include increasing incubation temperature, supplementing with essential nutrients and reactive oxygen species scavengers (e.g., sodium pyruvate) to neutralise stressors, and adding quorum sensing‐derived resuscitation‐promoting factors (Rpf) or autoinducer‐2 (AI‐2) (Chen et al. 2024).

Overall, the multilayered regulatory networks equip *Pseudomonas* with survival strategies and petroleum hydrocarbon degradation capabilities under varying types and intensities of environmental stress. In essence, the petroleum hydrocarbon degradation capacity and stress response in *Pseudomonas* are distinct yet interconnected from multiple perspectives. An effective degrading strain must possess robust stress adaptability, a trait dictated by its application scenarios and one that serves as a prerequisite for performing efficient bioremediation. Concurrently, the transcriptional regulatory mechanisms governing degradation and stress adaptation substantially overlap, centering on shared processes such as redox balance and energy homeostasis.

### 3.3 Significant Role of *Pseudomonas* in Actual Degradation Communities Revealed Based on Co‐Occurrences in the Microbiome

**FIGURE 4:**
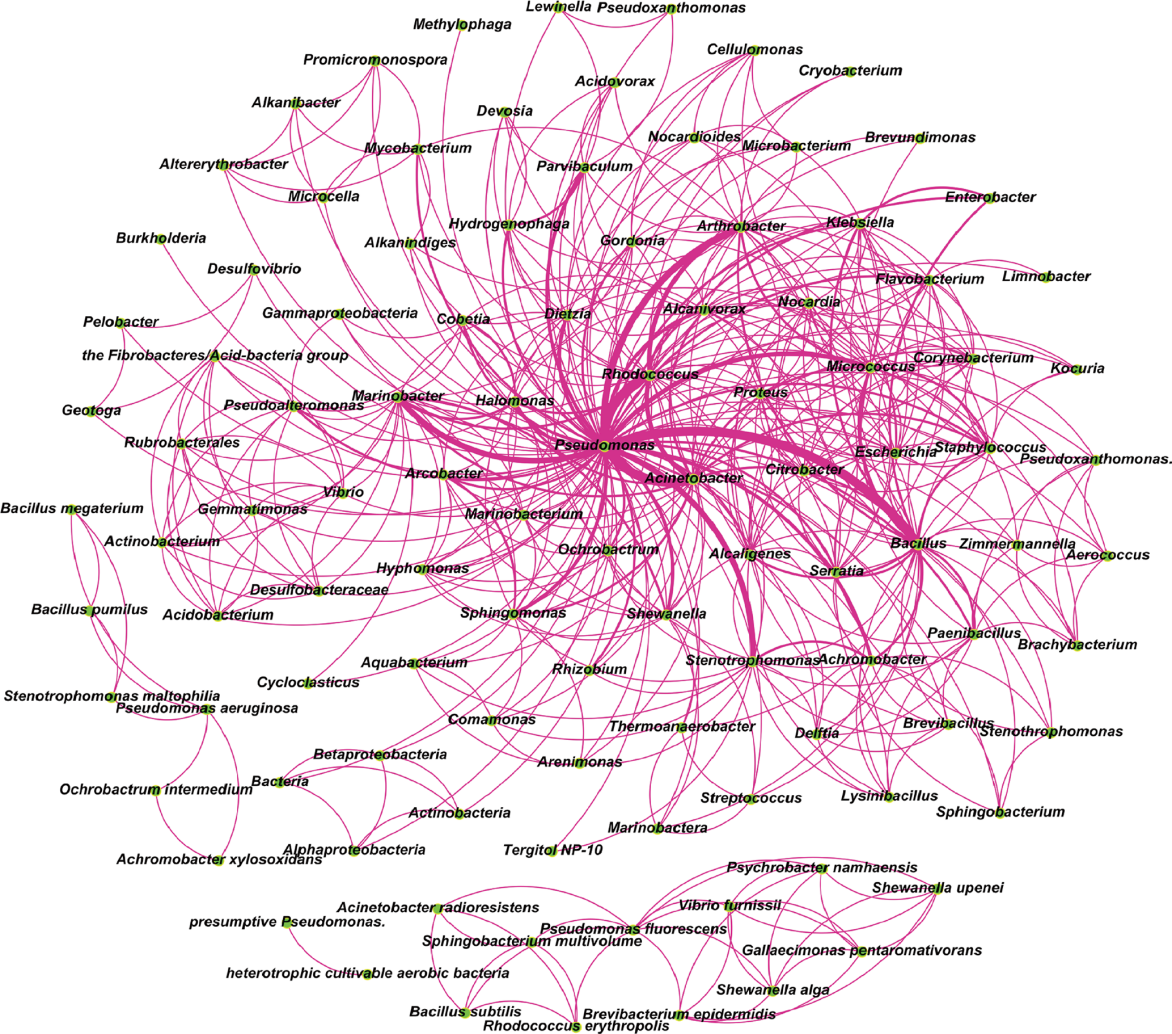
Significant role of *Pseudomonas* in actual degradation communities revealed based on co‐occurrence relationships in the microbiome. The co‐occurrence network of *Pseudomonas* and other bacteria was mapped using the stable degrading flora appearing in the 100 most cited literature in the past 10 years as the data source.

The above analysis revealed the excellent degradation capacity and broad substrate spectrum of *Pseudomonas* strains. To further elucidate their ecological role in practical bioremediation, the co‐occurrence network of *Pseudomonas* and other bacteria was mapped using the stable, high‐efficiency degrading consortia identified from the 100 most‐cited publications over the past decade. Figure 4 vividly shows the complex interactions of various petroleum hydrocarbon‐degrading bacteria, revealing *Pseudomonas* as the most central genus. Its highest co‐occurrence frequency was observed with *Pseudomonas, Bacillus*, *Rhodococcus*, *Acinetobacter*, *Marinobacter*, *Arthrobacter*, and *Stenotrophmonas*—all recognised as typical degraders. Besides *Pseudomonas*, the genera *Rhodococcus* and *Acinetobacter* also occupy central positions in the co‐occurrence network, reflecting their wide degradation spectra and stress adaptability. For example, a consortium composed of *Pseudomonas* sp. and *Rhodococcus erythropolis* achieved over 60% TPH removal within 15 days (Perdigao et al. 2021). While *Rhodococcus* and *Acinetobacter* are known for efficient alkane degradation (Milic et al. 2024; Liu et al. 2016), *Pseudomonas* bacteria have relatively outstanding degradation efficiency for alkanes and aromatic hydrocarbons. *Pseudomonas* also plays a key role in microbial‐phytoremediation by promoting plant growth and enhancing stress resistance (Dimkic et al. 2022). Its ability to produce metabolites like pyocyanin further improves its aggregation and dominance in crude oil environments (Rojo 2010). These traits collectively position *Pseudomonas* as a pivotal genus shaping the structure and function of petroleum hydrocarbon‐degrading communities (Zhou et al. 2023). Therefore, deepening the understanding of species‐specific traits and genetic determinants within *Pseudomonas* is essential for optimising its compositional structure in synthetic communities, ultimately enabling more efficient and tailored bioremediation strategies.

### 3.4 Comparison of Distribution Patterns of Petroleum Hydrocarbon Degradation‐Related Genes Among Strains From Multiple *Pseudomonas* Species

While prior research has reported variations in degradation properties among *Pseudomonas* species, comprehensive cross‐species genomic comparisons are still lacking. To bridge this knowledge gap, we analysed whole‐genome sequences from representative strains across multiple *Pseudomonas* species, integrating existing literature and database annotations to systematically delineate genes involved in petroleum hydrocarbon degradation. Within a comparative genomics framework, we then examined inter‐species differences in degradation traits and their underlying genetic basis.

**FIGURE 5:**
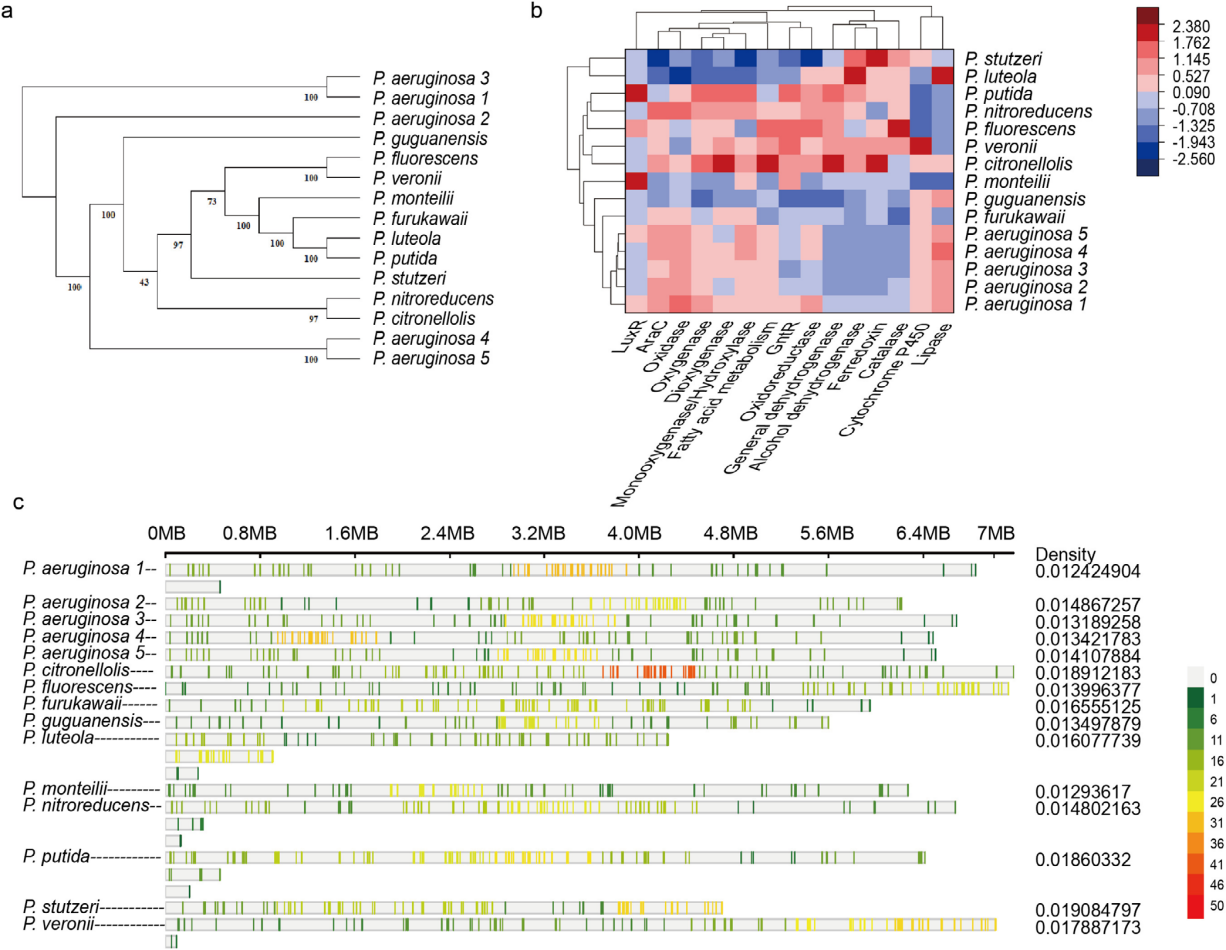
Comparison of distribution patterns of petroleum hydrocarbon degradation‐related genes among strains from multiple *Pseudomonas* species. (a) Phylogenetic trees of 15 representative *Pseudomonas* strains were constructed using their whole genome sequences. (b) Hierarchical clustering was performed based on the distribution patterns of various types of coding genes closely associated with petroleum hydrocarbon degradation among the strains. The criteria for identifying genes that are “associated with petroleum hydrocarbon degradation” are as follows: (1) genes encoding direct degrading enzymes; and (2) genes encoding transcriptional regulators that play a significant role in the degradation process. (c) Localisation and distribution of genes directly and indirectly related to biodegradation across the genomes of each *Pseudomonas* strain.

Based on whole‐genome sequences, an unrooted phylogenetic tree was constructed for 15 selected *Pseudomonas* strains to elucidate their evolutionary relationship (Figure 5a). Frequently reported strong degraders, *P. aeruginosa* and *P. putida* , fell into two distinct clades. *P. aeruginosa* strains 1 and 3 formed a separate monophyletic clade, while strains 2, 4, and 5 of the same species are clustered together in another basal group. *P. putida* , along with other species such as *P. veronii* , *P. luteola* , *P. monteilii* , *P. furukawaii*, *P. stutzeri* and *P. citronellolis* , forms branches after several nodes. This phylogenetic pattern reflects substantial genetic diversity among *Pseudomonas* species, suggesting that they may harbour different degradation functional profiles.

A comparative analysis of conserved genes associated with petroleum hydrocarbon degradation revealed distinct genetic patterns correlated with functional capabilities. *P. citronellolis* and *P. putida* possessed the largest repertoires of such genes. The encoded proteins include specific monooxygenases/hydroxylases, dioxygenases, alcohol dehydrogenases, cytochrome P450, ferredoxins, key regulatory proteins, and enzymes in downstream fatty acid metabolism. Some proteins could only be annotated at broad functional levels, such as general oxygenases and dehydrogenases, or even as general oxidases and oxidoreductases.

It reflects the diverse catalytic strategies employed by these strains. Clustering based on the distribution of degradation‐related gene categories revealed potential functional groupings (Figure 5b). Notably, five *P. aeruginosa* strains clustered together, characterised by elevated counts of genes encoding AraC, monooxygenases/hydroxylases, general oxidases, general oxygenases, cytochrome P450, lipases, and catalytic enzymes associated with fatty acid metabolism. This genetic consistency corresponds to their observed efficiency in degrading aliphatic hydrocarbons, such as *n*‐hexadecane and *n*‐decane (Stancu 2022), supported by the high copy number of key genes like *alkB* and *rhlAB* (Dong et al. 2017). The conserved genetic architecture among these *P. aeruginosa* strains highlights a relatively stable degradation foundation within the species. In strains of *P. citronellolis* , *P. nitroreducens* , *P. putida* , and *P. veronii* , we observed relatively high numbers of genes encoding monooxygenases/hydroxylases, GntR, general oxygenases, dioxygenases, general oxidoreductases, general dehydrogenases, and alcohol dehydrogenases. Species‐specific enrichments were also evident: LuxR‐family regulators in *P. monteilii* , AraC‐family regulators and general oxidases in *P. nitroreducens* ; GntR‐family regulators, general oxidoreductases, and catalases in *P. fluorescens*; genes encoding cytochrome P450 in *P. veronii*; lipases in *P. luteola* ; and ferredoxins in *P. stutzeri* . These variations imply a potential labor division and cooperation during petroleum hydrocarbons degradation (Liu et al. 2024).

The genomic locations and distribution of genes directly and indirectly associated with biodegradation were mapped across 15 strains (Figure 5c). Genome sizes vary considerably ranging from 4,273,365 to 7,309,421 bp. In terms of the proportion of degradation‐related genes relative to the total genes, *P. citronellolis* has the highest ratio at 20.995%. Although some strains (e.g., *P. aeruginosa* ) possess a considerable number of degradation‐related genes, their proportions are lower, indicating richness in other functional genes. Such strains exhibit strong degradation potential and versatility. For instance, even under nutrient‐limited conditions, *P. aeruginosa* WD23 was able to degrade 27.25% of petroleum crude oil within 15 days and tolerated salinity levels of up to 4.0% in mass‐to‐volume ratios (Goveas et al. 2020). A considerable proportion of degrading genes are densely distributed throughout the genome, which is conducive to the co‐expression and interaction among these genes, thereby enhancing rapid response to pollutants. In *P. putida* AK5, genes encoding degradation‐related enzymes are typically located in the same operon (Izmalkova et al. 2013), while genes in *P. aeruginosa* DN1 associated with substrate recognition, binding transport, and biodegradation metabolic processes function coordinately (Li, Tian, et al. 2020). Most degradation genes were located on chromosomes rather than plasmids, which may relate to epigenetic regulation of the degradation process via nuclear chromatin conformation (Tian et al. 2020).

To validate the genetic basis of degradation traits identified through literature and annotation, a genome‐wide conserved domain analysis was performed on *P. aeruginosa 1*, which harbours numerous degradation‐related genes. Key conserved domains included: Enoyl‐CoA hydratase (ECH superfamily), epoxide hydrolase superfamily, flavin monooxygenase superfamily, NAD (P)‐dependent oxidoreductase superfamily, cytochrome P450 monooxygenase superfamily, etc. (Figure S2). The enzymes and proteins encoded by these genes play crucial roles in various petroleum hydrocarbon degradation steps. The abundance of genes encoding monooxygenase/hydroxylase, general oxidase, general oxygenase, cytochrome P450 and lipase in *P. aeruginosa* aligns with its recognised capacity to metabolise long‐chain alkanes and aromatics via evolutionarily conserved oxygenases (Medic and Karadzic 2022). The conserved domain profile reflects the strain's strong degradation capability and supports the reliability of our comparative genomic analysis. Both genomic characteristics and previously reported experimental evidence underscore the complexity and diversity of degradation gene composition across *Pseudomonas* species, firmly linking genetic diversity to functional adaptability.

### 3.5 Petroleum Hydrocarbon Degradation Characteristics of Strains and Their Potential Complementarity Highlighted by Core and Accessory Genes

**FIGURE 6:**
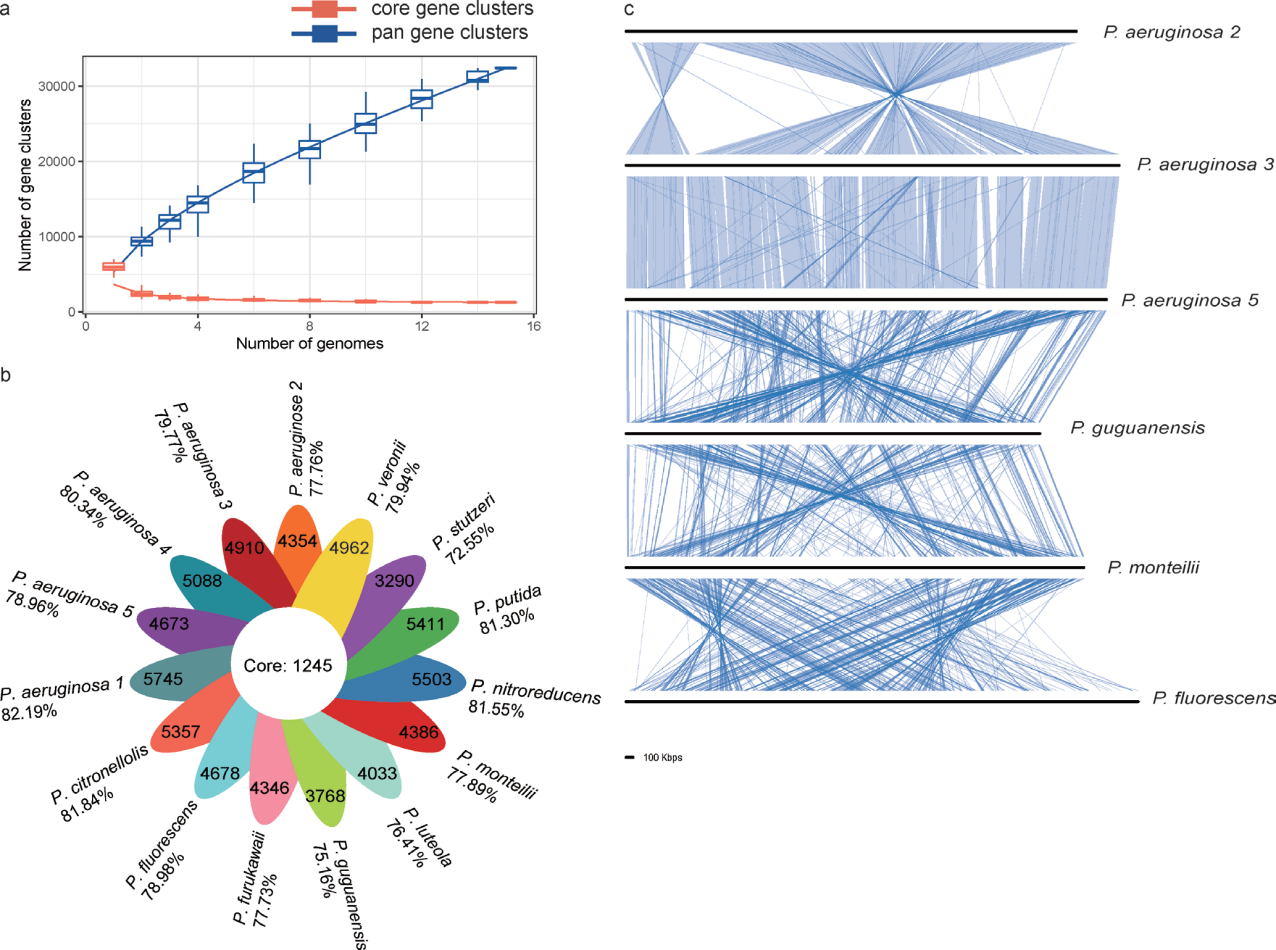
Distribution of core and accessory genes in the genomes of investigated petroleum hydrocarbon‐degrading *Pseudomonas* strains. (a) the trend in the number of co‐occurring core genes and total genes as different strains are added. (b) the distribution of the number of accessory genes, excluding core genes, across different strains. (c) Significant collinearity present in certain genomic regions of partial strains.

To further explore the degradation traits and potential synergies among strains, this study innovatively employed a pan‐genome approach to analyse and compare published genomes from 15 *Pseudomonas* species. This method summarises the intersection and union of gene sets across strains, with intersections pointing to orthologous gene clusters. After removing redundancy based on orthology, the cumulative gene count of 15 *Pseudomonas* strains increased steadily as more genomes were included (Figure 6a), indicating substantial expansion of gene diversity with each added strain. The first eight genomes contributed an average of 2838 genes each, while subsequent additions increased the total by approximately 1398 genes per genome, culminating in a final tally of 32,449 genes across all 15 strains. In contrast, the number of shared core genes decreased sharply upon inclusion of the second strain, and then stabilised. There are 1245 core genes in 15 *Pseudomonas* strains, accounting for about 20% of all genes in each strain. The number of non‐core (accessory) genes varied substantially among strains, ranging from 3290 to 5745, accounting for 72.55% to 82.19% of the total (Figure 6b). Overall, these results reveal relatively limited core genes and extensive genetic diversity among *Pseudomonas* strains from different species.

Certain *Pseudomonas* strains exhibit high genomic collinearity in specific regions, as depicted in Figure 6c. Strains including *P. aeruginosa* 2, *P. aeruginosa* 3, *P. aeruginosa* 5, *P. guguanensis*, *P. monteilii* , and *P. fluorescens* show well‐conserved gene order and physical distribution in these segments, indicating structural preservation across species. Collinearity is most pronounced among *P. aeruginosa* 2, *P. aeruginosa* 3, and *P. aeruginosa* 5, although a clear transposition event is observed between *P. aeruginosa* 2 and *P. aeruginosa* 3 at the genome level. This structural conservation likely reflects their common species origin. In contrast, eight other strains display low overall collinearity and are omitted from the figure, underscoring substantial genomic rearrangement variability among diverse *Pseudomonas* strains. Core gene and collinearity analyses collectively reveal significant genetic individuality across *Pseudomonas* species. A key ensuing question is what functional phenotypes are associated with shared versus unique genes, and whether petroleum hydrocarbon degradation genes predominantly belong to the core or accessory gene pool.

**FIGURE 7:**
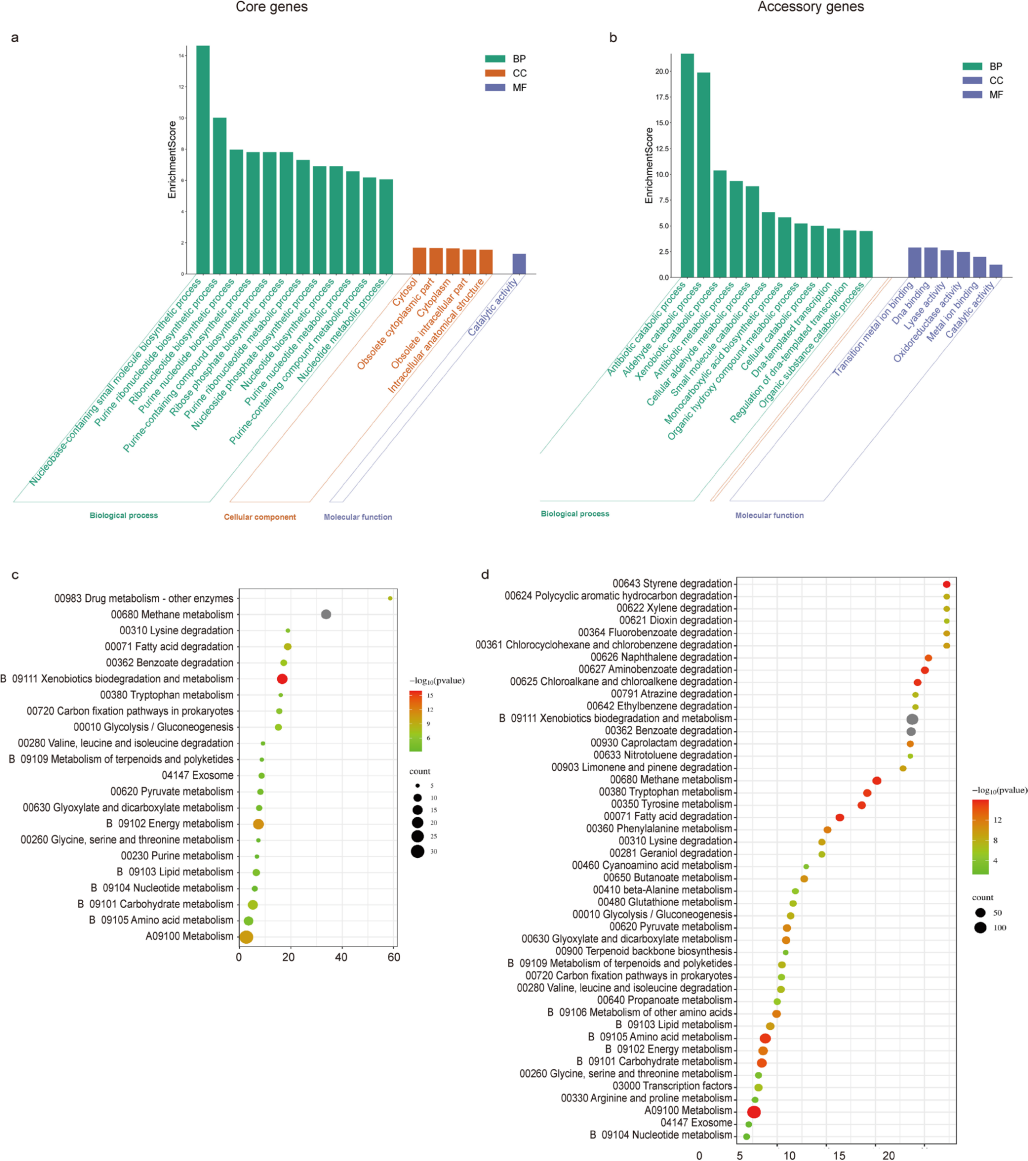
GO and KEGG enrichment analysis of co‐occurring core genes and accessory genes among 15 *Pseudomonas* strains. The core genes are primarily enriched in categories related to fundamental cellular physiology and biochemistry (a), while the accessory genes are enriched in categories associated with degradation processes (b). KEGG analysis indicates that basal metabolic pathways are enriched in the core gene set (c), while pathways related to xenobiotic biodegradation and metabolism are enriched in the accessory gene set (d).

To elucidate the association between genetic commonality/individuality and petroleum hydrocarbon degradation, we conducted GO and KEGG enrichment analyses on core and accessory genes across 15 *Pseudomonas* strains. As shown in Figure 7a, GO enrichment revealed that core genes are primarily involved in fundamental cellular processes, maintaining basic physiological activities of *Pseudomonas*. In contrast, various degradation‐related processes—such as antibiotic catabolism, aldehyde catabolism, and xenobiotic catabolism—were predominantly enriched within the accessory gene set (Figure 7b). KEGG annotation further supported this pattern (Figure 7c,d): core genes were primarily involved in basal metabolic pathways including amino acid, nucleotide, and lipid metabolism, whereas accessory genes were markedly enriched in xenobiotic biodegradation pathways, such as styrene degradation (FDR = 1.11E‐16, Enrichment Factor = 22.26), xylene degradation (FDR = 7.56E‐09, Enrichment Factor = 22.26), and polycyclic aromatic hydrocarbon degradation (FDR = 7.56E‐09, Enrichment Factor = 22.25).

Overall, the enrichment analyses indicate that degradation‐related functions are primarily attributed to the accessory genes rather than the core gene. It highlights the considerable genetic diversity of *Pseudomonas* species in petroleum hydrocarbon degradation. This diversity may manifest as preferences for different substrate types or variations in metabolic pathways across strains. For example, *P. aeruginosa* demonstrates efficient degradation of medium‐ to long‐chain alkanes and aromatic hydrocarbons, growing robustly on *n*‐dodecane, *n*‐octadecane, benzene, toluene, xylene or naphthalene as the only carbon source (Liu et al. 2016). *P. putida* , *P. citronellolis* , *P. nitroreducens* and *P. veronii* are mostly emphasised to have outstanding ability to degrade polycyclic aromatic hydrocarbons (Ghorbannezhad et al. 2022; Singh and Fulekar 2010). Despite their similar broad degradation spectra, the distribution of degradation‐related genes differs between species, which means that functional complementarity and genetic diversity among *Pseudomonas* strains may underlie their synergistic roles in microbial consortia for enhanced biodegradation.

### 3.6 Synergism of Strains From Different *Pseudomonas* Species in Coping With Refractory Petroleum Hydrocarbon Components

Although direct evidence of metabolic complementarity among conspecific strains remains limited, insights can be gleaned from the metabolic characteristics of different strains. For example, the co‐culture of *P. nitroreducens* S8 and *P. monteilii* S17 has been shown to synergistically degrade crude oil derivatives (Yang et al. 2023). Specific enzymatic reactions reveal further distinctions: *P. aeruginosa* produces abundant long‐chain alkane hydroxylases (Li, Pan, and Ma 2020); *P. plecoglossicida* S7 secretes lipases tolerant to multiple organic solvents (Choudhary et al. 2023); *P. psychrophila* S2TR‐14 generates toluene/o‐xylene monooxygenase ToMO and catechol 1, 2‐dioxygenase C1, 2D for degradation of aromatic hydrocarbons (Miri et al. 2021). Meanwhile, *P. putida* PaW1 harbours genes encoding dehalogenase and 2, 5‐dichloro‐2, 5‐cyclohexadiene‐1, 4‐diol dehydrogenase involved in γ‐hexachlorocyclohexane degradation (Leahy et al. 2003). Based on previous research and our aforementioned findings, we further explored specific degradation pathways to visually present how *Pseudomonas* strains from different species collaborate in degrading complex organic pollutants at a genome‐wide scale.

**FIGURE 8:**
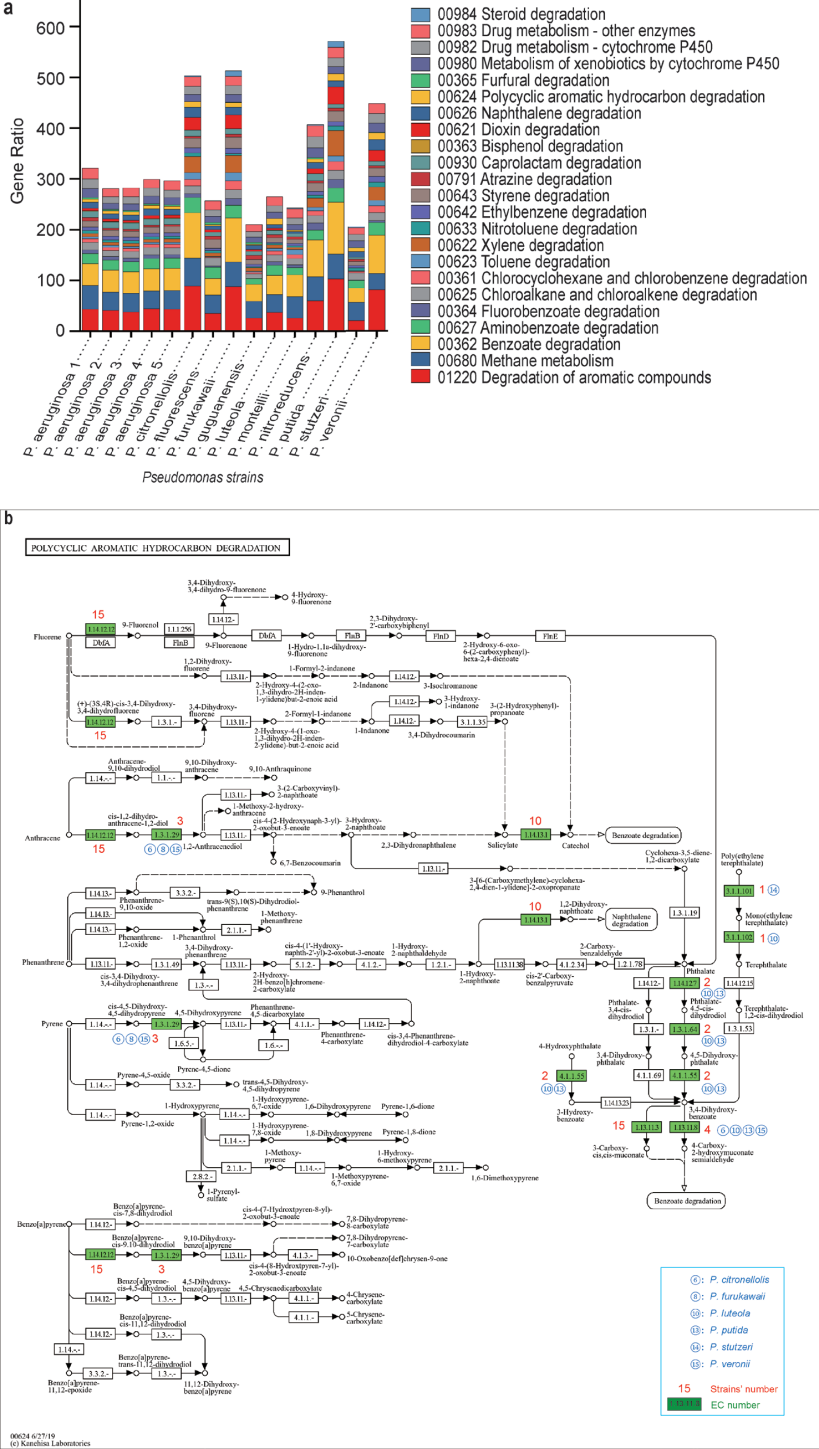
Synergism of strains from different *Pseudomonas* species in coping with refractory aromatic hydrocarbon components. (a) The stacked diagram illustrates the enrichment pattern of genes from various strains in the KEGG pathway related to the biodegradation of aromatic compounds. (b) The PAH degradation pathway is populated with genes responsible for PAH degradation across different *Pseudomonas* strains, thereby highlighting the contributions of these strains to the integrity of the PAH degradation pathway. This figure was annotated using the KEGG Mapper platform. In our study, a “node” represents a specific enzymatic reaction, defined by its Enzyme Commission (EC) number (green colour), the red numbers marked above the green box represent the number of strains containing this node.

Aromatic hydrocarbons, noted for their structural stability and well‐annotated degradation pathways in the KEGG database, were chosen for in‐depth analysis. A genome‐wide comparison showed that *P. putida* , *P. furukawaii*, *P. citronellolis* , *P. veronii* , and *P. nitroreducens* contained the highest number of genes enriched in aromatic compound degradation pathways (Figure 8a). Bibliometric analysis further highlighted the proficiency of these five strains in this regard. At the same time, this data is largely consistent with the genome‐wide analysis on degradation‐related genes, indicating that *P. putida* , *P. citronellolis* , *P. veronii* , and *P. nitroreducens* possess a more comprehensive repertoire of degradation enzymes and regulatory proteins. Specifically, *P. putida* showed the strongest gene enrichment in dioxin degradation (00621) and xylene degradation (00622) pathways; *P. aeruginosa* has the largest number of genes enriched to the xenobiotic degradation involving cytochrome P450 (00980); *P. aeruginosa* and *P. luteola* each possessed genes for bisphenol degradation pathway (00363); and styrene degradation (00643) was prominent in *P. citronellolis* , *P. fluorescens* , *P. furukawaii* and *P. putida* . Among conspecific strains, five *P. aeruginosa* strains displayed similar gene numbers and proportions across aromatic compound degradation, reaffirming a conserved genetic basis for biodegradation within species. In addition, most pathways were represented—albeit to varying degrees—across the studied strains, supporting their functional relevance.

For polycyclic aromatic hydrocarbons (PAHs), a recalcitrant subclass of pollutants, we mapped PAH degradation genes from different *Pseudomonas* strains onto the canonical KEGG pathway to evaluate their collective coverage. Figure 8b illustrates that the genes of *P. aeruginosa* , *P. luteola* and *P. putida* exhibited the strongest complementary gene distribution across pathway nodes. Enzymes from these strains collectively addressed critical steps in PAH degradation, including initial oxidation by dioxygenases (e.g., NahAc in *P. putida* ), intermediate cleavage by catechol dioxygenases (e.g., CatA in *P. aeruginosa* ), and further processing of aromatic intermediates by hydrolases and dehydrogenases (e.g., FdhA in *P. luteola* ). This enzymatic division of labor minimises metabolic bottlenecks and expands the substrate range of the consortium. For instance, *P. aeruginosa* contributes robust initial oxidation capabilities, *P. putida* ensures efficient intermediate channelling, and *P. luteola* enhances the mineralisation of persistent metabolites. Such synergy is further reinforced by the coordinated expression of surfactant genes (e.g., *rhlA, lasAB*) that improve substrate bioavailability.

The complementary genetic elements distributed across different *Pseudomonas* strains provide a genomic blueprint for designing efficient degradative consortia. Guided by this blueprint, we constructed a core consortium of *P. aeruginosa, P. luteola*, and *P. putida*. Leveraging the modular distribution of complementary genetic functions among *Pseudomonas* strains maximises pathway coverage and reaction efficiency, forming a basis for the rational design of synthetic degradative consortia.

### 3.7 Integrated Analysis Combining Bibliometrics, Comparative Genomics, and Pan‐Genome Approaches to Reveal Synergistic Degradation Potential of Multiple Strains

Innovatively, this study extends the pan‐genome concept from a single species to encompass the entire *Pseudomonas* genus. By integrating bibliometric analysis with comparative genomics, we systematically examined the degradation traits of diverse petroleum hydrocarbon‐degrading *Pseudomonas* strains at the molecular level. The results reveal both conserved and strain‐specific genetic features. In terms of gene content, the considerable diversity of degradation‐related genes among different *Pseudomonas* species suggests a potential for functional division of labor and cooperation during the degradation process. Genomically, while most degradation genes are located on the chromosome, a substantial proportion is distributed across the entire genome, which may facilitate gene co‐expression and functional interactions. Notably, a majority of the genes annotated with degradation functions belong to the accessory genome of each strain, highlighting how distinct metabolic capabilities have evolved in response to specific polluted environments. For instance, enzymes produced by different strains collectively cover more nodes in the degradation pathways of persistent pollutants like PAHs. This genetic complementarity suggests that different *Pseudomonas* strains can function synergistically within a community to enhance the metabolism of diverse petroleum hydrocarbon pollutants. Consequently, the inherent metabolic complementarity and co‐culture stability observed among *Pseudomonas* strains render them highly suitable for constructing synthetic microbial communities with enhanced biodegradation capabilities.

## 4 Conclusions and Prospects

In summary, *Pseudomonas* species show strong potential for bioremediation, underpinned by a robust genetic basis that includes key genes for surfactant biosynthesis (e.g., *rh1A, rh1B, rh1C, lasAB, lasR*) and catalytic enzymes (e.g., monooxygenases, dioxygenases, cytochrome P450). Multilevel regulatory networks further support their stress tolerance. Genus‐wide pan‐genome analysis reveals functional diversity and complementarity among strains, indicating that synergistic interactions can broaden the degradation spectrum and improve efficiency in pollutant removal. The stability and cooperative interactions observed in co‐culture systems underscore the advantage of multi‐strain consortia over single strains for complex degradation tasks.

The innovation of this study is threefold: First, extending pan‐genome analysis from a single species to the entire genus, leveraging the stable coexistence of closely related species; Second, demonstrating complementary crude oil‐degrading traits among *Pseudomonas* species by integrating genotypic and phenotypic evidence; and third, developing a genus‐centric strategy for constructing intergeneric consortia with superior synergistic efficacy.

These findings are expected to advance two areas: (1) Parallel strategies for constructing efficient biodegradation communities. By integrating top‐down and bottom‐up approaches and focusing on specific functional strains, community structures can be optimised with emphasis on genus‐level composition. The strong bioremediation capabilities, metabolic complementarity, and co‐culture stability of *Pseudomonas* strains make this genus an ideal hub for synthetic communities. The comparative genomics approach used here also provides a reference for applying reverse genetics in community design. (2) Acquisition of high‐quality gene elements for elite strain engineering. Combining metagenomics, comparative genomics, and phenotype–genotype association allows for the precise identification of key genes enhancing bioremediation across strains. Elucidating the genetic bases of degradation, metabolic complementarity, and stress responses supports targeted incorporation of crucial gene elements via genetic manipulation (He et al. 2023), enabling the creation of superior degraders with broad substrate ranges and robust stress tolerance.

## Author Contributions

**Xiaopeng Guo:** conceptualization, methodology, supervision, funding acquisition, writing – review and editing, validation, data curation. **Shuhua Zhu:** conceptualization, methodology, writing – original draft, writing – review and editing, software, data curation, visualization. **Ning Zhu:** investigation, software, project administration, visualization, writing – original draft. **Shuhan Zhang:** data curation, software, visualization. **Shenghui Yang:** investigation, validation, formal analysis, software. **Guanghong Luo:** methodology, validation, project administration. **Hongbin Li:** formal analysis, supervision, resources, writing – review and editing. **Yonggang Wang:** supervision, resources, writing – review and editing, methodology, funding acquisition. **Jing Sun:** funding acquisition, formal analysis, project administration. **Borong Ma:** project administration, investigation, formal analysis.

## Funding

This work was supported by the National Natural Science Foundation of China, 12205132; The 77th batch of general funding from China Postdoctoral Science Foundation, 2025MD774059; The Hexi University President Fund, QN2024011; The Gansu Province Joint Fund General Project, 25JRRA1148; The Lanzhou Science and Technology Plan Project, 2024‐3‐80; The Hongliu Excellent Youth Support Program in Lanzhou University of Technology, Fourth Batch.

## Ethics Statement

The authors have nothing to report.

## Consent

The authors have nothing to report.

## Conflicts of Interest

The authors declare no conflicts of interest.

## Supporting information


**Figure S1:** The transcriptional regulation mechanisms in *Pseudomonas* strains often inducted by multiple signal molecules, such as the genes that encode degrading enzymes themselves, rhizosphere microecological factor, limiting nutrient elements, etc. Meanwhile, it is also affected by quorum sensing systems and REDOX reactions.
**Figure S2:** For *P. aeruginosa* 1, a bacterium possessing a significant number of genes related to degradation, a genome‐wide investigation into conserved domains was conducted to confirm the accuracy of the genetic foundations linked to its degradation capabilities.
**Table S1:** Genbank accession number and genome data acquisition address of *Pseudomonas* strains.
**Table S2:** *Pseudomonas*‐derived biosurfactants: functional classification and efficacy in petroleum hydrocarbon degradation.

## Data Availability

The data supporting the findings of this study can be found in the relevant databases as specified in the Methods section, with details also provided in the Supporting Information of this article.
