# Supplementary material for: Pan‐Genomic and Phenotypic Characterisation of Petroleum Hydrocarbon Degradation by Pseudomonas Species
Source: Environ Microbiol Rep. 2026 Feb 15;18(1):e70300. doi: 10.1111/1758-2229.70300 (PMC12907034; doi:10.1111/1758-2229.70300)
Supplement: Supplementary file 1 — Figure S1: The transcriptional regulation mechanisms in Pseudomonas strains often inducted by multiple signal molecules, such as the genes that encode degrading enzymes themselves, rhizosphere microecological factor, limiting nutrient elements, etc. Meanwhile, it is also affected by quorum sensing systems and REDOX reactions. Figure S2: For P. aeruginosa 1, a bacterium possessing a significant number of genes related to degradation, a genome‐wide investigation into conserved domains was conducted to confirm the accuracy of the genetic foundations linked to its degradation capabilities. Table S1: Genbank accession number and genome data acquisition address of Pseudomonas strains. Table S2: Pseudomonas‐derived biosurfactants: functional classification and efficacy in petroleum hydrocarbon degradation. [file EMI4-18-e70300-s001.docx]

**Supporting Information**

**Pan-Genomic and Phenotypic Characterization of Petroleum Hydrocarbon Degradation by *Pseudomonas* Species**

Xiaopeng Guo^1, †^, Shuhua Zhu^1, †^, Ning Zhu^1^, Shuhan Zhang^1^, Shenghui Yang^2^, Guanghong Luo^2, *^, Hongbin Li^1, *^, Yonggang Wang^1, *^, Jing Sun^3^, Borong Ma^3^

^1^ School of Life Science and Engineering, Lanzhou University of Technology, Lanzhou 730050, China

^2^ Gansu Kaiyuan Biotechnology Development Center Co., Ltd, Zhangye 734000, China

^3^ Gansu Lanfei Environmental Protection Technology Co., Ltd, Lanzhou 730100, China

^*^Corresponding authors: E-mail: [lihongbin@lut.edu.cn](mailto:lihongbin@lut.edu.cn); [wangyg@lut.edu.cn](mailto:wangyg@lut.edu.cn)

**Content list**

Figure S1: The transcriptional regulation mechanisms in *Pseudomonas* strains when degrading petroleum.

Figure S2: Analysis of conserved domains of *P. aeruginosa* 1.

Table S1: Download links of the selected *Pseudomonas* genome data on the NCBI.

Table S2: A five-category classification of biosurfactants from *Pseudomonas* strains: Functional roles and efficacy in petroleum hydrocarbon degradation.

**
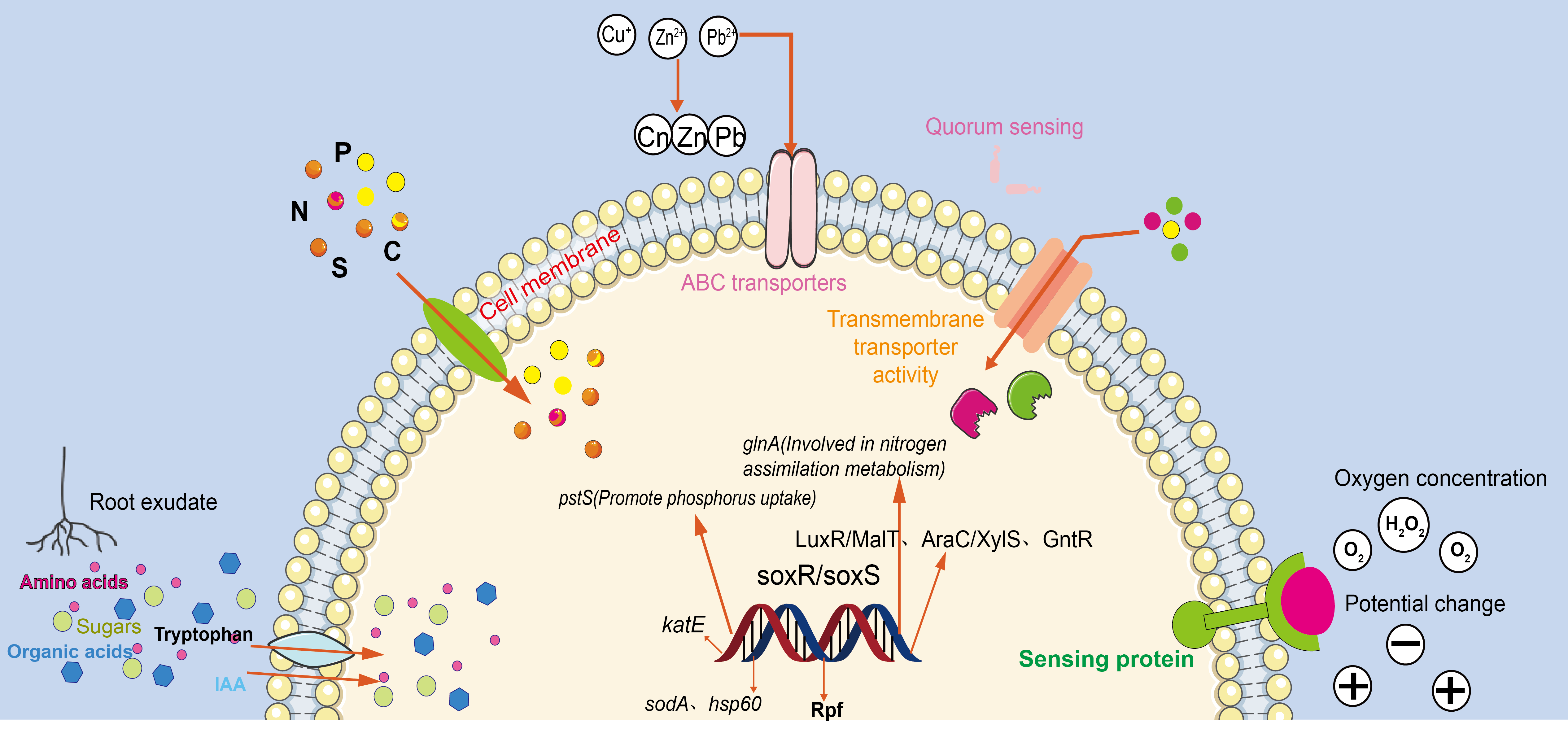
**

**Figure S1.** The transcriptional regulation mechanisms in *Pseudomonas* strains often inducted by multiple signal molecules, such as the genes that encode degrading enzymes themselves, rhizosphere microecological factor, limiting nutrient elements, etc. Meanwhile, it is also affected by quorum sensing systems and REDOX reactions.


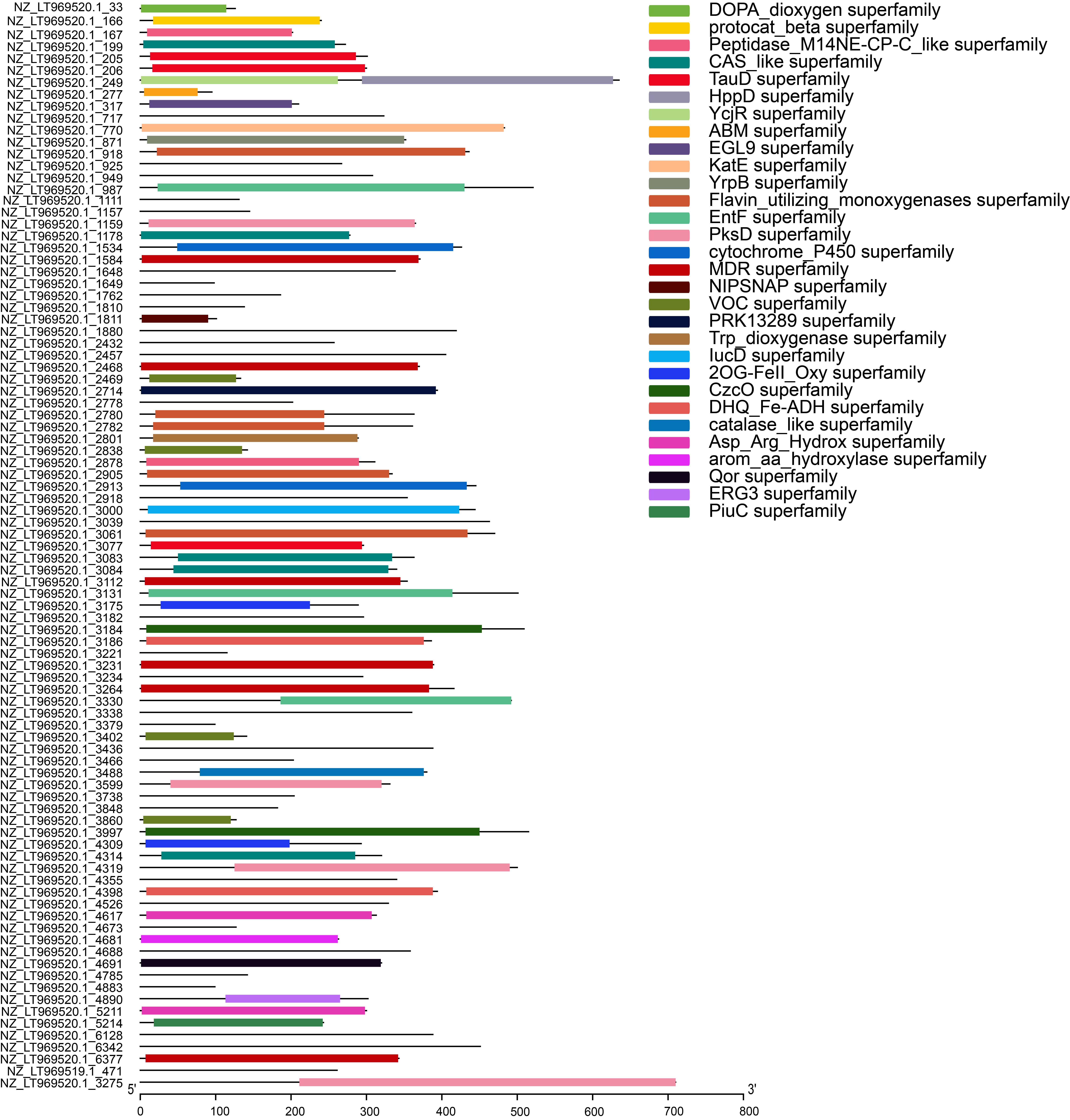


**Figure S2.** For *P. aeruginosa* 1, a bacterium possessing a significant number of genes related to degradation, a genome-wide investigation into conserved domains was conducted to confirm the accuracy of the genetic foundations linked to its degradation capabilities.

**Table S1.** Genbank accession number and genome data acquisition address of *Pseudomonas* strains.

| *Pseudomonas* strains | GenBank | NCBI Link | Abundance of Degradation Gene Clusters |
| --- | --- | --- | --- |
| *Pseudomonas aeruginosa* strain DN1 | GCA_001722005.2 | [*Pseudomonas aeruginosa genome assembly ASM172200v2 - NCBI - NLM (nih.gov)*](https://www.ncbi.nlm.nih.gov/datasets/genome/GCF_001722005.2/) | 321 |
| *Pseudomonas aeruginosa Isolate* RW109 | GCA_900243355.1 | [*Pseudomonas aeruginosa genome assembly RW109 - NCBI - NLM (nih.gov)*](https://www.ncbi.nlm.nih.gov/datasets/genome/GCF_900243355.1/) | 281 |
| *Pseudomonas aeruginosa* SJTD-1 | GCA_000271985.2 | [*Pseudomonas aeruginosa SJTD-1 genome assembly ASM27198v2 - NCBI - NLM (nih.gov)*](https://www.ncbi.nlm.nih.gov/datasets/genome/GCF_000271985.2/) | 282 |
| *Pseudomonas aeruginosa* Strain ATCC 27853 | GCA_024507955.1 | [*Pseudomonas aeruginosa genome assembly ASM2450795v1 - NCBI - NLM (nih.gov)*](https://www.ncbi.nlm.nih.gov/datasets/genome/GCF_024507955.1/) | 299 |
| *Pseudomonas aeruginosa* Strain L10 | GCA_002223805.1 | [*Pseudomonas aeruginosa genome assembly ASM222380v1 - NCBI - NLM (nih.gov)*](https://www.ncbi.nlm.nih.gov/datasets/genome/GCF_002223805.1/) | 296 |
| *Pseudomonas citronellolis* Strain SJTE-3 | GCA_001654435.1 | [*Pseudomonas citronellolis genome assembly ASM165443v1 - NCBI - NLM (nih.gov)*](https://www.ncbi.nlm.nih.gov/datasets/genome/GCF_001654435.1/) | 503 |
| *Pseudomonas fluorescens* Strain FW300-N2C3 | GCA_001307275.1 | [*Pseudomonas fluorescens genome assembly ASM130727v1 - NCBI - NLM (nih.gov)*](https://www.ncbi.nlm.nih.gov/datasets/genome/GCF_001307275.1/) | 257 |
| *Pseudomonas furukawaii* Strain KF707 | GCA_002355475.1 | [*Pseudomonas furukawaii genome assembly ASM235547v1 - NCBI - NLM (nih.gov)*](https://www.ncbi.nlm.nih.gov/datasets/genome/GCF_002355475.1/) | 513 |
| *Pseudomonas guguanensis* Strain HMFL31 | GCA_030323645.1 | [*Pseudomonas guguanensis genome assembly ASM3032364v1 - NCBI - NLM (nih.gov)*](https://www.ncbi.nlm.nih.gov/datasets/genome/GCF_030323645.1/) | 210 |
| *Pseudomonas luteola* Strain FDAARGOS_637 | GCA_008693965.1 | [*Pseudomonas luteola genome assembly ASM869396v1 - NCBI - NLM (nih.gov)*](https://www.ncbi.nlm.nih.gov/datasets/genome/GCF_008693965.1/) | 265 |
| *Pseudomonas monteilii* Strain NMI135_16 | GCA_021283055.2 | [*Pseudomonas monteilii genome assembly ASM2128305v2 - NCBI - NLM (nih.gov)*](https://www.ncbi.nlm.nih.gov/datasets/genome/GCF_021283055.2/) | 243 |
| *Pseudomonas nitroreducens* Strain HBP1 | GCA_011044415.1 | [*Pseudomonas nitroreducens genome assembly ASM1104441v1 - NCBI - NLM (nih.gov)*](https://www.ncbi.nlm.nih.gov/datasets/genome/GCF_011044415.1/) | 407 |
| *Pseudomonas putida* Strain KF715 | GCA_002356095.1 | [*Pseudomonas putida genome assembly ASM235609v1 - NCBI - NLM (nih.gov)*](https://www.ncbi.nlm.nih.gov/datasets/genome/GCF_002356095.1/) | 571 |
| *Pseudomonas stutzeri* (*Stutzerimonas stutzeri* Strain F2) | GCA_018138085.1 | [*Stutzerimonas stutzeri genome assembly ASM1813808v1 - NCBI - NLM (nih.gov)*](https://www.ncbi.nlm.nih.gov/datasets/genome/GCF_018138085.1/) | 252 |
| *Pseudomonas veronii* Strain Pvy | GCA_004919535.4 | [*Pseudomonas veronii genome assembly ASM491953v4 - NCBI - NLM (nih.gov)*](https://www.ncbi.nlm.nih.gov/datasets/genome/GCF_004919535.4/) | 205 |

**Table S2.** *Pseudomonas*-derived biosurfactants: functional classification and efficacy in petroleum hydrocarbon degradation.

| **Biosurfactants** | **Representative *Pseudomonas* Strains** | **Surface Tension Reduction** | **Solubilization & Emulsification** | **Antimicrobial Activity & Environmental Adaptability** | **Degradation Efficiency & Mechanism** | **References** |
| --- | --- | --- | --- | --- | --- | --- |
| Glycolipids- Rhamnolipids | *P. aeruginosa* M4; *P. aeruginosa* SARSHI2 | 30 mN/m (CMC: 0.1 g/L) | E24: 63.75%; Crude oil emulsification (120 h) | Halotolerant (70 g/L NaCl); Optimal temp. 35℃ | Oil degradation: 85.20%; Wax degradation: 53.16% | (Rashedi et al., 2015) |
| Lipopeptides- Surfactin | *P. fluorescens* SBW25; *P. putida* KT2440 | 28 mN/m (CMC: 40 mg/L) | E24: 81.16% (18 d stable); Liquid paraffin emulsification: 41.25% | pH 4-9 stable; Antibacterial against Gram+ bacteria | Crude oil degradation: 87.25%; Viscosity reduction: 32.24% | (Eslami et al., 2020) |
| Glycolipids- Sophorolipids | *P. aeruginosa* SS1; *P. sp.* KR3 | 32 mN/m (pH 2-8 stable) | E24: 72% ± 0.23; Crude oil dispersion: 70% | Heat-resistant (40℃); Heavy metal tolerance (1-15 mM Pb²⁺) | Acenaphthene degradation: 91.78% (21 d); C18-C24 alkane degradation | (Rahman et al., 2002) |
| Lipopeptides- Iturin | *P. aeruginosa* B-1; *P. sp.* L-1 | 32.19 mN/m (initial 65.74 mN/m) | Wax removal: 42.00%; Emulsion stability > 14 d | Cell hydrophobicity: 0.677; Optimal temp. 30℃ | Waxy crude oil degradation: 42.7%; Benz(a)fluorene degradation: 97.5% | (Jacoby et al., 2024) |
| Fatty Acid Derivatives | *P. sp.* MZ01; *P. koreensis* YS-314 | 30 mN/m (CMC: 0.1 g/L) | Light hydrocarbon (C10-C16) emulsification: 55% | Salt-tolerant (5% NaCl); pH 9.0 optimal | Crude oil degradation: 54.7% (9 d); n-Hexadecane removal: 84.7% | (Sen, 2010) |

**References**

1. Sharma, R., Singh, J. & Verma, N. 2018. Optimization of rhamnolipid production from *Pseudomonas aeruginosa* PBS towards application for microbial enhanced oil recovery. *3 Biotech*, 8, 20. https://doi.org/10.1007/s13205-017-1022-0
2. Eslami, P., Hajfarajollah, H., & Bazsefidpar, S. 2020. Recent advancements in the production of rhamnolipid biosurfactants by *Pseudomonas aeruginosa*. *RSC Adv.*, 10(56), 34014-34032. https://doi.org/10.1039/d0ra04953k
3. Rahman, K.S.M., Rahman, T.J., McClean, S., Marchant, R. and Banat, I.M. 2002. Rhamnolipid Biosurfactant Production by Strains of *Pseudomonas aeruginosa* Using Low-Cost Raw Materials. *Biotechnol. Prog.*, 18, 1277-1281. https://doi.org/10.1021/bp020071x
4. Jacoby, M.R., Dening, M.E. & Raiger Iustman, L.J. 2025. Simultaneous Production of Lipopeptide and Rhamnolipid Biosurfactants by *Pseudomonas aeruginosa*: A Promising Blend for Biosurfactant-Enhanced Bioremediation. *Curr. Microbiol.*, 82, 543. https://doi.org/10.1007/s00284-025-04526-1
5. Sen, R. 2020. Biosurfactants (ISBN 978‑1441959782). Bioprocess and Bioproduct Development Laboratory, Department of Biotechnology, Indian Institute of Technology Kharagpur, West Bengal, India. Springer.
